# National trends and county-level geographic disparities in mortality from operationally defined cardiovascular–kidney–metabolic stages 4 and 4b in the United States

**DOI:** 10.3389/fcvm.2026.1838375

**Published:** 2026-05-29

**Authors:** Kaide Xia, Junwen Wang, Bingpeng Gao

**Affiliations:** 1 Guiyang Maternal and Child Health Care Hospital, Guiyang Children’s Hospital, Guiyang, Guizhou, China; 2 Department of Psychosomatic Medicine, The Second People’s Hospital of Guiyang, Guiyang, China; 3 Department of Urology, Zhejiang Provincial People's Hospital Bijie Hospital (The First People's Hospital of Bijie), Bijie, China

**Keywords:** cardiorenal overlap, CKM syndrome, geographic inequities, joinpoint regression, kidney failure involvement, mortality

## Abstract

**Introduction:**

Advanced cardiovascular–kidney–metabolic (CKM) syndrome includes stage 4, defined by clinical cardiovascular disease in the CKM context, and stage 4b, defined as stage 4 with kidney failure. Long-term mortality trends and geographic inequities for these advanced stages are not well characterized.

**Methods:**

We performed an ecological time-series and spatial analysis of U.S. death certificate data from the CDC WONDER Multiple Cause of Death database, 1999–2023. Using an operational death-certificate–based proxy, stage 4 was defined by clinical cardiovascular disease codes, and stage 4b as stage 4 with concurrent renal failure (N17–N19). We calculated annual age-adjusted mortality rates (AAMRs), estimated annual percent change (APC) and average annual percent change (AAPC) using joinpoint regression, and mapped 2023 state- and county-level rates and derived metrics, including Stage 4b share within Stage 4. County clustering was assessed using Local Moran's I.

**Results:**

From 1999 to 2023, age-adjusted mortality declined for stage 4 from 505.54 to 353.30 per 100,000 population (average annual percent change, −1.31%; 95% confidence interval, −1.52 to −1.10) and for stage 4b from 45.95 to 33.98 per 100,000 (−1.18%; −1.75 to −0.34). However, trends reversed in the past decade: stage 4 increased after 2013 (annual percent change, +1.17%), whereas stage 4b increased after 2015 (+4.63%). Stage 4 mortality formed a high-burden belt across the Deep South and Appalachia, whereas stage 4b hotspots were more dispersed. Stage 4b share showed corridor-like clustering across parts of the Great Plains and Mountain West.

**Conclusion:**

Mortality from operationally defined advanced CKM stages declined overall but rebounded in the past decade, with substantial geographic inequities. Surveillance that tracks both absolute burden and the kidney failure–associated component of advanced CKM mortality may help target integrated cardio-renal risk reduction strategies.

## 1 Introduction

Cardiovascular–kidney–metabolic (CKM) syndrome has emerged as a unifying framework capturing the intertwined pathophysiology and clinical consequences of obesity, diabetes, chronic kidney disease (CKD), and cardiovascular disease (CVD) (1–4). The American Heart Association (AHA) presidential advisory introduced a staging construct (Stages 0–4) for CKM syndrome, in which Stage 4 represents the highest-risk stage characterized by clinical CVD in the context of CKM conditions (2, 5). Stage 4 is further subdivided into Stage 4a (without kidney failure) and Stage 4b (with kidney failure), reflecting distinct clinical complexity and management considerations (1, 6). Despite the conceptual importance of Stage 4—particularly Stage 4b, which captures co-occurring clinical CVD and kidney failure—population-level evidence on long-term mortality trends and geographic inequities for these advanced stages remains limited (5, 7–9). Because CKM Stage 4/4b represents potentially preventable trajectories of cardiometabolic risk progression and care fragmentation, documenting when-and-where mortality reversals concentrate can inform targeted prevention, implementation strategies, and health-system planning beyond national averages.

Concurrently, prior work has documented that the decades-long decline in United States (US) cardiovascular mortality slowed substantially in the early 2010s, with deceleration in the rate of decline after approximately 2011 (10–12). Such macro-trend shifts may translate into distinct patterns for advanced CKM mortality rates, with potentially heterogeneous impacts across demographic subgroups and local health systems, shaped by structural and place-based determinants of health (13–16). However, few studies have combined long time horizons with national, state, and county resolution to identify inflection points and spatial clustering in advanced CKM mortality (5).

To address these gaps, we examined US mortality rates for CKM Stage 4 and Stage 4b from 1999 to 2023 using national vital statistics multiple-cause-of-death data. We quantified temporal patterns using joinpoint regression to estimate segment-specific annual percent change (APC) and overall average annual percent change (AAPC), evaluated heterogeneity by sex, age group, race, urbanization, and Census region, and characterized state- and county-level geographic disparities. We also assessed the relationship between Stage 4 and Stage 4b using a rate ratio (Stage 4/Stage 4b), the corresponding share (Stage 4b/Stage 4), and the absolute rate difference (Stage 4−Stage 4b), and quantified county-level spatial clustering using Local Indicators of Spatial Association (LISA).

## 2 Methods

### 2.1 Data source

We conducted an ecological time-series and spatial analysis of US mortality data from 1999 to 2023 using the Centers for Disease Control and Prevention (CDC) Wide-ranging ONline Data for Epidemiologic Research (WONDER) Multiple Cause of Death database and standardized extracts derived from it (17–19). The database compiles information from death certificates for United States residents and provides counts, age-adjusted rates, standard errors/95% confidence intervals, and both underlying and contributing (multiple) causes of death by geography and demographic strata. This study used aggregated, de-identified public data and did not involve human participants; institutional review board review was not required.

### 2.2 CKM stage 4/4b ascertainment

We operationalized CKM staging according to the AHA CKM framework. Stage 4 corresponds to clinical CVD in the context of CKM syndrome and is subdivided into Stage 4a (without kidney failure) and Stage 4b (with kidney failure) (1). Using US death certificate data, we identified Stage 4 and Stage 4b from ICD-10 codes recorded as underlying and/or contributing causes of death (multiple-cause fields). Stage 4 was defined by the presence of any of the following CVD codes: I20–I25 (ischemic heart diseases), I50 (heart failure), I60–I69 (cerebrovascular diseases), I48 (atrial fibrillation and flutter), or I70 (atherosclerosis) (20). Stage 4b was defined as Stage 4 with concurrent kidney failure, identified by codes N17–N19 (renal failure) (20). In sensitivity analyses, we redefined kidney failure using a more specific code set excluding acute renal failure (N17). The primary temporal and spatial patterns were generally similar under this alternative kidney failure definition.

The primary outcomes were annual age-adjusted mortality rates (AAMR) for Stage 4 and Stage 4b, expressed per 100,000 population and standardized to the 2000 US standard population when obtained from CDC WONDER outputs (21). For comparisons between Stage 4 and Stage 4b, we constructed (i) a ratio for trend modeling, r = AAMR_Stage4_/AAMR_Stage4b_, and (ii) a difference for trend modeling, d = AAMR_Stage4_−AAMR_Stage4b_. For descriptive mapping (state/county), we additionally used the share = AAMR_Stage4b_/AAMR_Stage4_ (the reciprocal of the ratio) to express the relative contribution of Stage 4b within Stage 4 (22, 23).

We analyzed national and subgroup trends stratified by sex (male and female), age group (non-elderly, 15–64 years, vs. elderly, ≥65 years), race (harmonized into three categories: White, Black, and Other for consistency across datasets), urbanization status (Metro vs. Nonmetro), and US Census region (Northeast, Midwest, South, and West).

### 2.3 Trend analysis and uncertainty estimation

When standard errors (SEs) were not provided, we approximated SEs from 95% confidence intervals (CIs) using: SE≈(Upper−Lower)/(2 × 1.96). For the ratio r, we used a delta-method approximation on the log scale: $SE_{\log\;(r)}=\sqrt{{\left(\frac{SE_{4}}{Rate_{4}}\right)}^{2}+{\left(\frac{SE_{4b}}{Rate_{4b}}\right)}^{2}}$, with 95% CI computed as $\exp(\log\;(r)\pm 1.96\times SE_{\log\;(r)})$. For the difference d, we used: $SE_{d}=\sqrt{SE_{4}^{2}+SE_{4b}^{2}},\;CI=d\pm 1.96\times SE_{d}$. Joinpoint regression was applied to annual AAMR time series to identify calendar years in which mortality trends changed and to estimate segment-specific annual percent change (APC) and the overall average annual percent change (AAPC), with corresponding 95% confidence intervals (24). In this context, APC represents the estimated yearly percentage change within a specific time segment, whereas AAPC summarizes the average yearly percentage change across the entire study period. The models were fitted using a log-linear form, which assumes that rates change by a constant percentage within each segment rather than by a constant absolute amount. We permitted a maximum of two joinpoints to balance flexibility with interpretability and to reduce overfitting in annual time-series data. For strata with differing data availability, the models were fitted over the range of years for which data were available. Because joinpoint trend models were fit on a log scale, the modeled outcome must be positive; if the difference metric contained non-positive values within a location series, we applied a within-location constant shift [adding |min (d)| + ɛ] to enable log-linear modeling; shifted series were explicitly labeled in outputs.

### 2.4 Spatial analysis

For 2023, we mapped state-level AAMR for CKM Stage 4 and Stage 4b and state-level derived metrics (share and gap). To examine long-term changes, we constructed state time series for 1999–2023 by concatenating 2 query outputs split at 2018 (years <2018 and  ≥2018) and fit joinpoint models by state to estimate state-level AAPCs. For AAPC maps, we used a diverging scale centered at 0 and applied common limits based on the maximum absolute AAPC across Stage 4 and Stage 4b.

For county-level analyses, we used tabular datasets containing deaths and population by 5-year age groups. We excluded strata above age 84 years (85–89, 90–94, 95–99, and  ≥100 years) and calculated county AAMR for ages 15–84 years using direct standardization to the 2000 US standard population restricted to ages 15–84. Because CDC WONDER suppresses small cell counts to protect confidentiality and reduce instability, suppressed death counts were handled using midpoint substitution, assigning deaths = 5 for suppressed cells. This value represents the midpoint of the usual suppressed range for counts below 10 and allows standardized county-level rates to be estimated consistently across geographic units.

This approach may introduce measurement error, especially in sparsely populated counties or strata with very low death counts. Midpoint substitution may overestimate rates if the true suppressed count is closer to 1 and underestimate rates if the true count is closer to 9. Therefore, county-level estimates based on suppressed cells should be interpreted as approximate, particularly for small counties. To evaluate the influence of this assumption, we repeated county-level rate estimation, decile mapping, and LISA analyses using alternative suppressed-count assignments of deaths = 1 and deaths = 9. These sensitivity analyses showed that Stage 4 AAMR was relatively stable across assumptions, whereas Stage 4b AAMR and Stage 4b share were more sensitive to the assumed suppressed-count value, particularly under the deaths = 1 scenario. Accordingly, we interpreted county-level Stage 4b and share-based findings cautiously, focusing on broad spatial patterns rather than precise county-specific estimates. County-level metrics were mapped using deciles (Q1–Q10), and the county-level share (Stage 4b/Stage 4) was used as the primary mapped indicator. We assessed local spatial autocorrelation using Local Moran I (LISA) (25). Analyses were restricted to the contiguous United States and to counties with nonmissing values and total population (ages 15–84 years) ≥ 20,000. We constructed queen contiguity neighbors and row-standardized spatial weights. For Stage 4 and Stage 4b AAMR, we applied a log transformation before LISA; share was analyzed without transformation. Counties were classified as high-high, low-low, high-low, or low-high, or as not significant or excluded. Statistical significance was prespecified at *P* ≤ 0.05. Given the large number of local tests in LISA, cluster maps were interpreted as hypothesis-generating indicators of place-based inequities rather than definitive evidence of statistically confirmed clusters.

## 3 Results

### 3.1 National and stratified temporal trends

From 1999 to 2023, national age-adjusted mortality rates (AAMR) declined for both CKM Stage 4 and Stage 4b. Stage 4 AAMR decreased from 505.54 per 100,000 in 1999 to 353.30 per 100,000 in 2023, and Stage 4b AAMR decreased from 45.95 to 33.98 per 100,000 (Table 1; Supplementary Table S1). Over the full period, declines were statistically significant, with an average annual percent change (AAPC) of −1.31 (95% CI, −1.52 to −1.10) for Stage 4 and −1.18 (95% CI, −1.75 to −0.34) for Stage 4b (Table 1).

**Table 1:** AAPC in national and subgroup AAMR for CKM stage 4 and stage 4b, 1999–2023.

Characteristics	Stage 4 AAPC (95% CI)	Stage 4b AAPC (95% CI)
Overall	−1.31 (−1.52 to −1.1)	−1.18 (−1.75 to −0.34)
Sex		
Female	−1.53 (−1.73 to −1.33)	−1.31 (−1.91 to −0.48)
Male	−1.43 (−1.73 to −1.09)	−1.31 (−1.84 to −0.52)
Age		
Elderly	−1.48 (−1.68 to −1.27)	−1.29 (−1.88 to −0.41)
Non-elderly	−0.65 (−1.02 to −0.37)	−0.43 (−1.09 to 0.47)
Race		
Black	−1.79 (−2.14 to −1.49)	−1.83 (−2.40 to −1.10)
Other	−2.10 (−2.42 to −1.81)	−2.79 (−3.34 to −1.94)
White	−1.19 (−1.39 to −0.99)	−1.00 (−1.59 to −0.15)
Urbanization^a^		
Metro	−1.65 (−1.94 to −1.48)	−2.27 (−2.94 to −1.40)
Nonmetro	−1.07 (−1.29 to −0.94)	−1.67 (−2.34 to −0.89)
Census regions		
Midwest	−1.18 (−1.38 to −0.98)	−1.21 (−1.73 to −0.52)
Northeast	−1.90 (−2.09 to −1.70)	−1.85 (−2.38 to −1.17)
South	−1.19 (−1.43 to −0.94)	−0.92 (−1.49 to −0.08)
West	−1.29 (−1.50 to −1.08)	−1.01 (−1.61 to −0.17)

a AAPCs for Urbanization strata were estimated for 1999–2020

AAPC, average annual percent change; AAMR, age-adjusted mortality rate; CKM, cardiovascular–kidney–metabolic.

**Figure 1:**
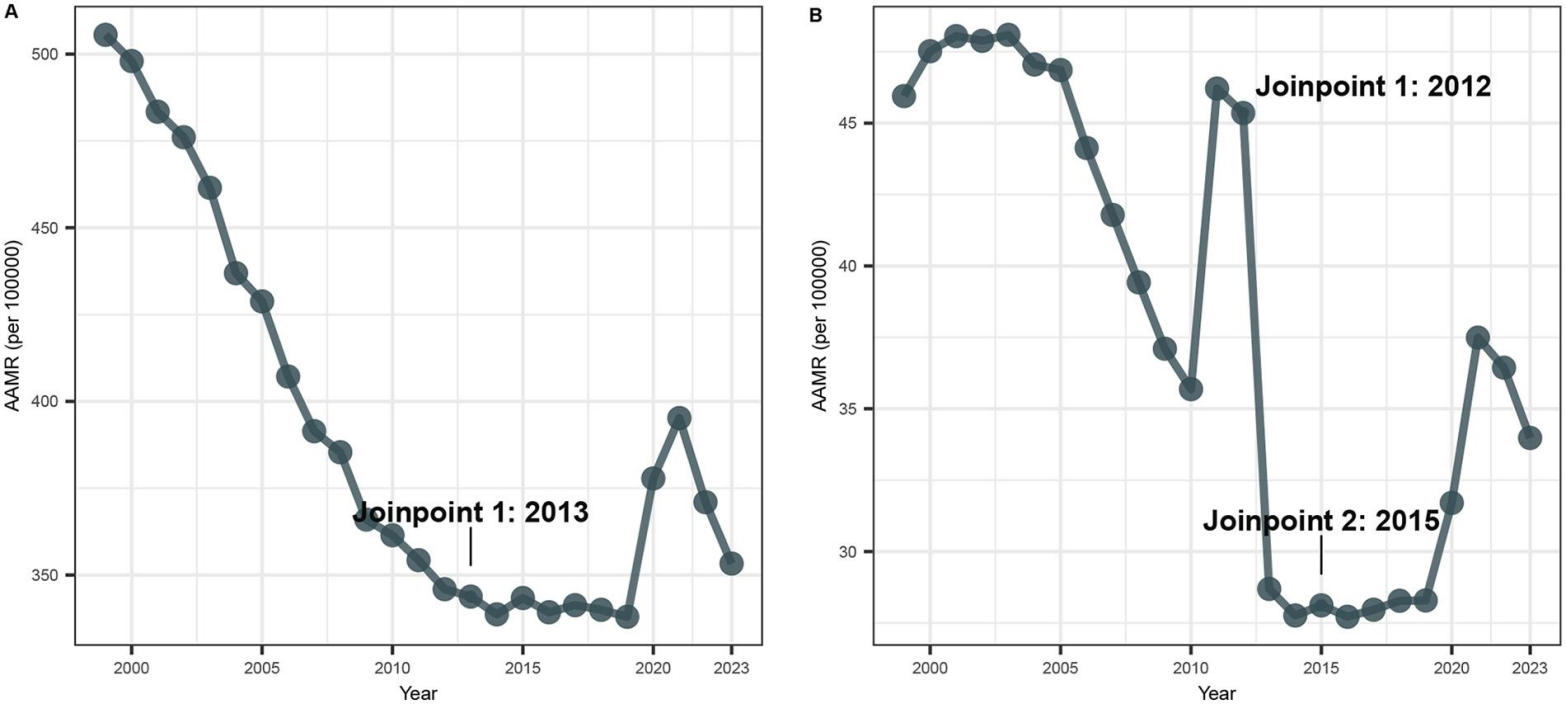
Joinpoint trends in national AAMR for CKM stage 4 (**A**) and stage 4b (**B**), 1999–2023. Points represent observed annual AAMRs, and fitted line segments represent joinpoint regression estimates. Joinpoints indicate calendar years in which the slope of the mortality trend changed. AAPC, average annual percent change; AAMR, age-adjusted mortality rates; CKM, cardiovascular–kidney–metabolic.

Joinpoint analyses indicated that major inflection points clustered around 2012–2015, with an additional inflection for Stage 4 around 2013. For Stage 4, AAMR declined significantly during 1999–2013 (APC, −3.05; *P* < .001) and then increased during 2013–2023 (APC, + 1.17; *P* = .002). For Stage 4b, the trend during 1999–2012 was not significant (APC, −1.53; *P* = .13), followed by a sharp decline during 2012–2015 (APC, −13.80; *P* = .006) and a subsequent increase during 2015–2023 (APC, + 4.63; *P* = .006) (Figure 1; Supplementary Table S2).

Long-term declines were generally observed across most subgroups, although their magnitude varied. Stage 4 declines were slightly faster among females than males (AAPC, −1.53 vs. −1.43), whereas Stage 4b declined at a similar rate in both sexes (AAPC, −1.31 for each) (Table 1; Supplementary Figure S1). Declines were more pronounced in the elderly (≥65 years) group (Stage 4 AAPC, −1.48; Stage 4b AAPC, −1.29) than in the non-elderly (15–64 years) group (Stage 4 AAPC, −0.65; Stage 4b AAPC, −0.43) (Table 1; Supplementary Figure S2). By race, Stage 4 decreased most rapidly in the Other category (AAPC, −2.10), followed by Black (−1.79) and White (−1.19) populations (Table 1; Supplementary Figure S3). Declines were larger in metropolitan than nonmetropolitan areas (Stage 4: −1.65 vs. −1.07; Stage 4b: −2.27 vs. −1.67; urbanization series available through 2020), and the Northeast exhibited the greatest reductions (Stage 4: −1.90; Stage 4b: −1.85) (Table 1; Supplementary Figures S4, 5).

Overall, Stage 4 and Stage 4b showed broad long-term declines across strata, with inflection points commonly occurring around 2012–2015 followed by stabilization or rebound. The non-elderly group showed the smallest long-term declines and less distinct post-2012 segmentation than other strata (Supplementary Figures S1–5; Supplementary Table 2), suggesting relative stagnation compared with older adults and other subgroups.

### 3.2 Trends in stage 4–stage 4b relationship

**Figure 2:**
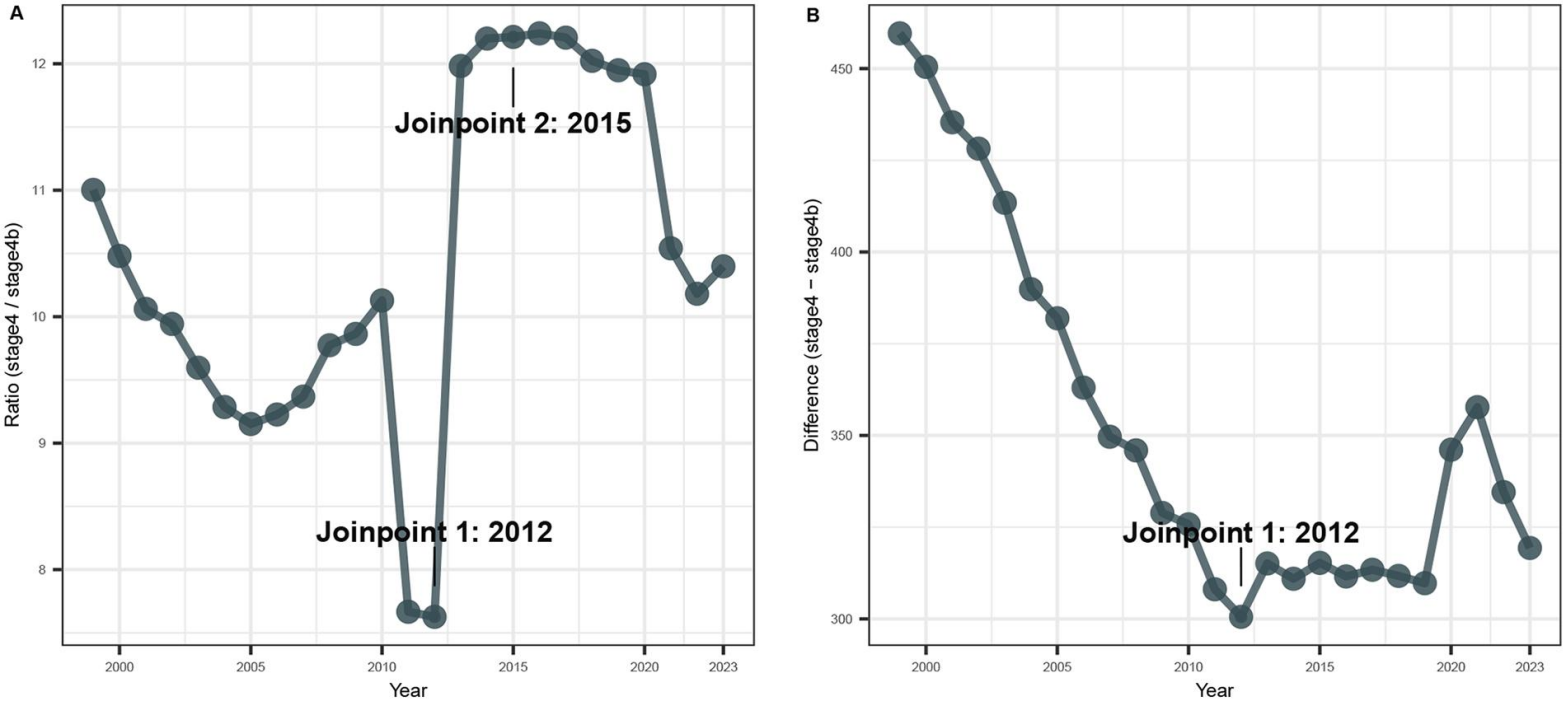
Joinpoint trends in the relationship between CKM stage 4 and stage 4b mortality, United States, 1999–2023. (**A**) shows the ratio of Stage 4 AAMR to Stage 4b AAMR, (**B**) shows the absolute difference between Stage 4 AAMR and Stage 4b AAMR. The ratio was calculated as Stage 4 AAMR divided by Stage 4b AAMR, and the absolute difference was calculated as Stage 4 AAMR minus Stage 4b AAMR. The ratio reflects the relative relationship between the two mortality rates, whereas the difference reflects the absolute mortality-rate gap. AAMR, age-adjusted mortality rate; CKM, cardiovascular–kidney–metabolic.

Nationally, the Stage 4/Stage 4b ratio remained relatively stable over time (AAPC, −0.13; 95% CI, −0.74–0.34). In contrast, the absolute difference (Stage 4−Stage 4b) narrowed significantly (AAPC, −1.31; 95% CI, −1.50–−1.12) (Figure 2; Supplementary Table S3). Joinpoint models for both metrics identified inflection points concentrated around 2012–2015, with a decline before 2012 and greater stability or modest fluctuations thereafter (Figure 2; Supplementary Table S3).

Across strata, the ratio was generally stable, with most AAPC estimates near 0% and 95% CIs crossing 0; the Other race category showed a significant increase (AAPC, 0.89; 95% CI, 0.28–1.43) (Supplementary Table S3). Ratio APC patterns typically showed a decline during 1999–2012, a short-term increase during 2012–2015, and a renewed decline after 2015; the early decline was not significant in the non-elderly group and the Other race category, and the post-2015 decline in the urbanization-stratified series did not reach significance (Supplementary Table S4).

Conversely, the absolute difference decreased significantly in all strata, with negative AAPCs (approximately −0.70%–−2.10%); larger reductions were observed in the Other and Black racial groups and in the Northeast region (Supplementary Table S3). Difference APC patterns were broadly consistent across strata—typically a marked decline in earlier years followed by post-2012 stabilization or rebound—although some strata showed additional late-period variability (e.g., renewed decline in the Northeast during 2021–2023 and a short-term increase in the urbanization series during 2018–2020) (Supplementary Table S4; Supplementary Figures S6–10).

### 3.3 State-level geographic patterns

In 2023, substantial heterogeneity in AAMR was observed across states. Stage 4 AAMR ranged from 259.29 to 474.09 per 100,000, with higher rates concentrated in the South and Appalachia-adjacent region (e.g., Mississippi, Oklahoma, West Virginia, Arkansas, Kentucky) and lower rates in parts of the Northeast and Hawaii (Supplementary Table S5; Supplementary Figure S1^1^). Stage 4b AAMR ranged from 19.00 to 58.92 per 100,000, with higher values more prominent in portions of the Midwest/Great Plains and selected states (e.g., Kentucky, South Dakota, Nebraska, Wisconsin, Oregon) and lower values in states such as New Mexico, New York, Connecticut, Hawaii, and Arizona (Supplementary Table S6; Supplementary Figure S1^2^).

Derived metrics further highlighted contrasts between absolute burden and relative contribution. The AAMR share (Stage 4b/Stage 4) ranged from 0.052 to 0.129, with higher values in Kentucky, South Dakota, and Colorado and lower values in New Mexico, Louisiana, and New York (Supplementary Figure S13). The absolute gap (Stage 4−Stage 4b) ranged from 236.61 to 434.06 per 100,000 and largely mirrored the Stage 4 spatial distribution (Supplementary Figure S1^4^).

**Figure 3:**
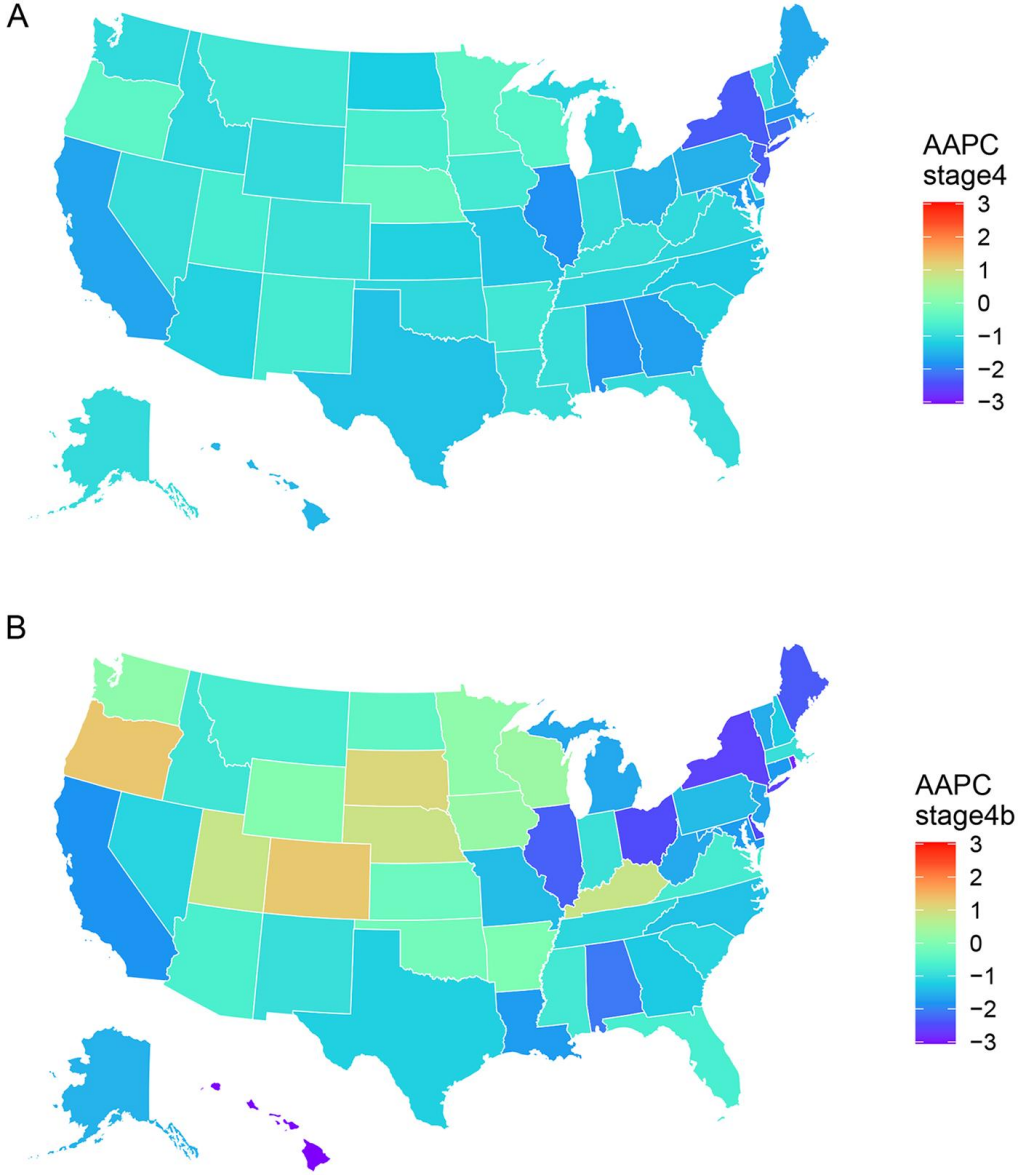
State-level AAPCs in AAMR for CKM stage 4 (**A**) and stage 4b (**B**), 1999–2023. Each state is colored according to the estimated AAPC in AAMR over the full study period. Negative values indicate declining mortality rates, whereas positive values indicate increasing mortality rates. A common diverging color scale centered at 0 was used to facilitate comparison between Stage 4 and Stage 4b. States with larger positive values represent areas with less favorable long-term trends. Map boundaries are shown for data visualization only and do not necessarily represent official boundaries or imply endorsement. AAPC: average annual percent change, AAMR, age-adjusted mortality rates; CKM, cardiovascular–kidney–metabolic.

From 1999 to 2023, state-level AAPCs for Stage 4 were negative for all states (approximately −2.33 to −0.32), with the largest reductions in the Northeast/Atlantic corridor (e.g., New York, New Jersey, Connecticut, and the District of Columbia) and smaller declines in states such as Nebraska, Minnesota, and Oregon (Supplementary Table S5). In contrast, Stage 4b trends were more heterogeneous: most states declined or showed no significant change, whereas several states showed significant increases (e.g., Oregon, Colorado, South Dakota, Nebraska, Kentucky, Utah) (Figure 3; Supplementary Table S6).

Segmented APC results highlighted key timing windows. For Stage 4, joinpoints commonly occurred around 2012–2014, followed by attenuated declines or transient rebound; some states showed an additional shift around 2021 (Supplementary Table S7). For Stage 4b, joinpoints were more consistently concentrated near 2012 and 2015, with pronounced short-term fluctuations during 2012–2015 and a more homogeneous phase of increase or rebound after 2015 (Supplementary Table S8). Together, these patterns suggest that Stage 4 declines were more uniform, whereas Stage 4b trajectories varied substantially across states, jointly shaping 2023 state differences in share and gap (Figure 3; Supplementary Tables S5–8; Supplementary Figures S11–14).

### 3.4 County-level patterns and spatial clustering

At the county level, Stage 4 AAMR showed marked spatial heterogeneity. High-decile counties formed a contiguous high-burden belt concentrated in the Deep South and Appalachia, whereas low-decile counties were more common in the Mountain West and the Northeast/New England region. In contrast, Stage 4b AAMR high-value counties were more fragmented and dispersed, with scattered concentrations across portions of the central and northern Plains in addition to localized clustering in the South (Supplementary Figure S15).

**Figure 4:**
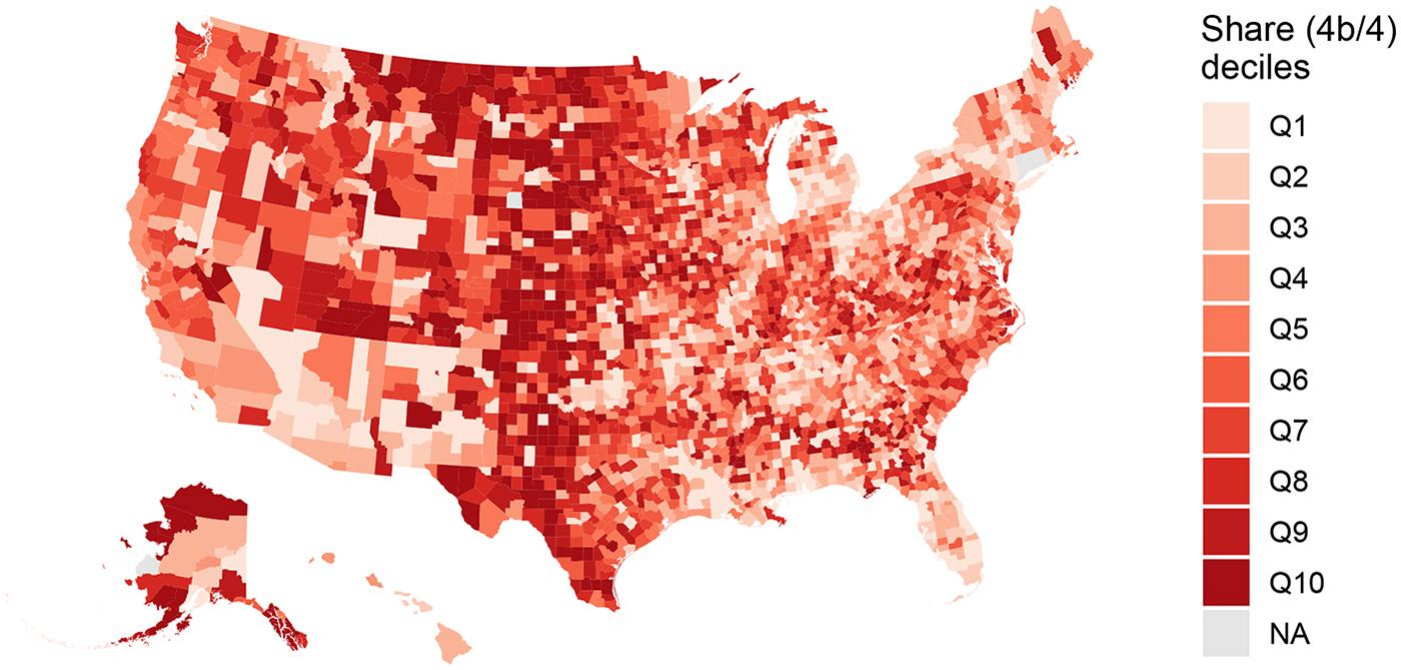
County-level distribution of the stage 4b share within CKM stage 4 mortality, United States, 2023. The share was calculated as Stage 4b AAMR divided by Stage 4 AAMR and represents the proportional contribution of kidney failure–associated deaths within operationally defined advanced CKM mortality. Higher values indicate a larger kidney failure–associated component within Stage 4 mortality. County values are displayed by deciles. Map boundaries are shown for data visualization only and do not necessarily represent official boundaries or imply endorsement. AAMR, age-adjusted mortality rate; CKM, cardiovascular–kidney–metabolic.

The county-level share (Stage 4b/Stage 4) exhibited a spatial gradient distinct from absolute AAMR, forming a north–south corridor from the Great Plains toward the Mountain West; lower-share counties were more common along the southeastern coastal areas and in parts of the Northeast (Figure 4). The county-level gap (Stage 4−Stage 4b) closely tracked the Stage 4 burden distribution, with higher-gap counties concentrated in the South and Appalachia (Supplementary Figure S16).

LISA analyses corroborated these patterns. High–High clusters for Stage 4 AAMR were concentrated in the Deep South and Appalachia, whereas Low–Low clusters were prominent in the Mountain West and parts of the Northeast. Stage 4b AAMR showed more spatially scattered hotspots. For share, High–High clusters extended across the Great Plains and parts of the Southwest, while Low–Low clusters were concentrated in portions of the Southeast coastal and Northeastern regions; many counties were not statistically significant for local spatial autocorrelation (Supplementary Figures S17, 18). Sensitivity analyses using alternative suppressed-count assignments indicated that Stage 4 AAMR and LISA patterns were relatively stable, whereas Stage 4b AAMR and Stage 4b share were more sensitive to the assumed value for suppressed cells. Nevertheless, the overall interpretation of substantial county-level heterogeneity and spatial clustering remained consistent (Supplementary Table S9).

## 4 Discussion

In this national, multi-scale assessment of advanced CKM mortality, we identified three key findings. First, from 1999 to 2023, AAMR for both CKM Stage 4 and Stage 4b declined overall, indicating a net reduction in mortality rates over the full study period. Second, this overall decline masked an important temporal reversal. Stage 4 mortality shifted from a marked decline during 1999–2013 to an increase during 2013–2023, whereas Stage 4b showed a brief rapid decline during 2012–2015 followed by a sustained increase after 2015. Third, geographic inequities were substantial and scale-dependent. Stage 4 mortality formed a contiguous high-burden belt in the Deep South and Appalachia, whereas Stage 4b patterns were more fragmented. The proportional contribution of Stage 4b within Stage 4 showed a distinct corridor-like geography across parts of the Great Plains and Mountain West, supported by statistically significant local clustering (4, 5, 26).

The early-2010s transition observed in advanced CKM mortality is temporally consistent with prior evidence that long-standing improvements in US cardiovascular mortality slowed around the same period (27, 28). Within the CKM framework, Stage 4 represents clinical CVD in the context of cardiometabolic and/or kidney conditions (1, 2, 7). The post-2013 increase in Stage 4 AAMR therefore suggests that earlier progress in reducing advanced cardiometabolic-cardiovascular mortality rates attenuated and reversed during the latter part of the study period (12, 15, 26, 28). This reversal may reflect the combined influence of worsening upstream cardiometabolic risk profiles and uneven access to effective prevention and chronic disease management. Persistent or increasing burdens of obesity, diabetes, hypertension, and CKD may have expanded the population at risk for advanced CKM phenotypes (1, 4, 29). In parallel, social determinants of health, rurality, and limited access to coordinated cardiovascular, metabolic, and kidney care may have reduced the population-level impact of preventive and therapeutic advances (12, 13, 30, 31).

Stage 4b showed a distinct segmentation pattern: a sharp decline during 2012–2015 followed by increases after 2015. Because Stage 4b reflects the co-occurrence of clinical CVD and kidney failure, the post-2015 increase may reflect changing dynamics in populations with the greatest clinical complexity (1, 2, 14). Potential contributors include shifts in the burden and severity of CKD and diabetes, delayed recognition of kidney disease, differences in access to nephrology and multidisciplinary cardio–renal care, and heterogeneity in kidney failure care pathways and cardiovascular risk management (2, 3, 7, 32–34). Importantly, the observed post-2015 increase should be interpreted as a combination of longer-term cardiometabolic trends and potential acute pandemic-related effects. The Stage 4b upward segment began in 2015, before the COVID-19 pandemic, suggesting that the rebound was not solely attributable to pandemic-related mortality. This pre-pandemic timing supports the possibility of an underlying cardiometabolic and cardio-renal trend, including increasing burden or severity of diabetes, CKD, and cardiovascular multimorbidity. However, the 2020–2023 portion of the segment may have been amplified by the COVID-19 pandemic through both direct and indirect pathways. Direct effects may include acute infection-related cardiovascular and kidney complications among high-risk individuals, whereas indirect effects may include delayed emergency care, disruption of routine chronic disease management, reduced preventive visits, medication interruptions, and strain on healthcare systems. Prior studies have reported excess cardiovascular mortality and disruption of cardiovascular care during the pandemic, supporting the need to interpret late-period increases cautiously (35, 36). Overall, these explanations are biologically and clinically plausible, but they should be interpreted as hypothesis-generating because the ecological design and death-certificate data cannot establish individual-level mechanisms or separate long-term CKM progression from acute COVID-19–related complications or pandemic-related healthcare disruptions.

Joint evaluation of Stage 4 and Stage 4b indicates that relative and absolute indicators provide complementary insights (37). Nationally, the Stage 4/Stage 4b ratio was relatively stable over time, suggesting limited long-term change in the proportional relationship between Stage 4 mortality rates and the subset with kidney failure. In contrast, the absolute gap narrowed over the full period, indicating decreasing absolute separation between Stage 4 and Stage 4b mortality rates. Together, these findings suggest that changes in advanced CKM mortality were not dominated by large national shifts in composition toward or away from kidney failure–associated deaths, even as absolute rates changed over time. Importantly, national stability in ratio-based measures does not preclude pronounced local heterogeneity (38). County share maps and LISA results demonstrate that “stable on average” can coexist with “highly uneven locally,” underscoring the importance of multi-scale surveillance and the limitations of relying only on national summary measures (39, 40).

Although declines were broadly observed, subgroup analyses indicated heterogeneity in the pace of improvement (29, 41). Larger long-term declines among older adults compared with non-elderly adults suggest that mortality reductions were not uniform across the life course. Several explanations may account for the smaller improvements observed among non-elderly adults. First, cardiometabolic risk factors such as obesity, diabetes, and hypertension increasingly affect adults before older age, potentially accelerating progression to clinical CVD and kidney complications during midlife (1, 2, 11, 29, 42). Several health-practice and healthcare-system factors may also contribute. Non-elderly adults may have lower perceived cardiovascular and kidney risk, less frequent routine screening, and more fragmented insurance or care continuity, which could delay detection and treatment of diabetes, CKD, hypertension, and subclinical CVD. Work-related barriers, lower engagement with preventive care, medication affordability, food environment, sedentary behavior, and psychosocial stress may further reduce opportunities for early risk modification (29, 42). These factors are important for future prevention because advanced CKM mortality in non-elderly adults represents premature loss of life and may foreshadow a larger future burden as affected cohorts age. Therefore, earlier-life CKM prevention, risk-factor screening, sustained primary care engagement, and integrated management of obesity, diabetes, hypertension, CKD, and CVD may be essential to prevent further stagnation or reversal in this group.

Differences by sex, race, region, and urbanization further suggest that advanced CKM mortality is shaped by heterogeneous exposures and health-system contexts. The slightly greater declines among females may reflect sex differences in baseline risk profiles, health-care utilization, competing causes of death, and treatment-seeking patterns, although death-certificate data cannot directly evaluate these mechanisms. Racial differences should also be interpreted cautiously because the broad race categories used in this analysis may mask substantial heterogeneity within groups; nevertheless, they are consistent with the broader literature showing that structural and place-based determinants influence cardiovascular, kidney, and metabolic outcomes. Less favorable trends in nonmetropolitan populations, noting that urbanization-stratified series were available through 2020, are consistent with persistent rural disadvantages in access to specialty care and fragmented chronic disease management—an issue highlighted as central to CKM care optimization (30, 31, 43, 44). Additionally, contemporaneous mortality trend shifts from non-cardiovascular comorbidities in older adults may influence the observed patterns in advanced multimorbidity constructs (45).

At the state level, Stage 4 AAPCs were uniformly negative, indicating broadly shared long-term declines. In contrast, Stage 4b trends were more heterogeneous, with several states showing significant long-term increases. This divergence is clinically plausible because Stage 4b specifically captures kidney failure superimposed on clinical CVD, which may be particularly sensitive to state-level differences in CKD burden, diabetes prevalence, cardiometabolic risk trajectories, dialysis and nephrology care availability, and access to integrated cardiovascular–kidney care (1, 2, 46, 47). Cross-sectionally in 2023, Stage 4 mortality rates concentrated in the South/Appalachia-adjacent region, reflecting entrenched geographic inequities in cardiometabolic and cardiovascular outcomes (29, 36). These areas have been repeatedly identified in prior cardiovascular mortality research as regions with higher cardiometabolic risk burden and structural barriers to prevention and treatment. Stage 4b's more dispersed geography suggests that kidney failure–associated advanced CKM mortality does not map perfectly onto the traditional cardiovascular “hotspot belt” (29). At the county level, the most distinctive spatial signal was the corridor-like distribution of share across parts of the Great Plains and Mountain West, reinforced by High–High LISA clustering (25, 43). This decoupling of “absolute burden” (Stage 4 AAMR) and “composition” (the proportional contribution of kidney failure within Stage 4) suggests that different local pathways may contribute to advanced CKM mortality. Accordingly, prioritization strategies may differ depending on whether the goal is to reduce overall advanced cardiovascular burden or to address disproportionate kidney failure involvement within advanced CKM mortality (47).

We examined 1999–2023 mortality using multiple-cause-of-death data and joinpoint models, integrating demographic stratification with state- and county-level mapping and LISA to characterize advanced CKM trends and geographic clustering. However, several limitations warrant consideration. First, this is an ecological analysis based on death certificate data. Misclassification, incomplete recording of contributing causes of death, and temporal changes in certification or coding practices may affect trends, particularly for multi-condition constructs such as advanced CKM mortality (48). Because the analysis was based on aggregated data, the observed temporal and geographic patterns cannot be linked to individual-level risk factors, treatment histories, or clinical trajectories, and no causal conclusions can be drawn. Second, CKM stage assignment from mortality data uses ICD-10 rule logic and cannot reproduce clinical staging based on laboratory measures, such as estimated glomerular filtration rate, albuminuria, blood pressure, glycemic status, body mass index, or other metabolic biomarkers; under- or over-identification is therefore possible (2). Our definitions operationalize CKM Stage 4/4b as death-certificate phenotypes intended for population surveillance rather than clinical staging based on biomarkers. Therefore, these findings should not be interpreted as directly classifying individual patients according to the full clinical CKM staging framework. Future studies linking mortality data with electronic health records, claims data, laboratory values, medication use, and individual-level risk factors are needed to validate these operational definitions and clarify mechanisms underlying the observed temporal and geographic patterns. Third, county-level AAMR was constructed for ages 15–84 years and included midpoint substitution for suppressed counts to enable aggregation. These necessary choices may affect comparability to all-age estimates and introduce measurement error in sparsely populated counties (3, 19). In particular, assigning deaths = 5 to suppressed cells assumes that the true value lies near the midpoint of the suppressed range; this may overestimate or underestimate rates when the actual count is closer to the lower or upper bound. In sensitivity analyses using alternative assignments for suppressed cells, Stage 4 AAMR and LISA classifications were relatively stable, whereas Stage 4b AAMR and Stage 4b share were more sensitive to the assumed suppressed-count value, particularly when suppressed cells were assigned deaths = 1. Therefore, county-level Stage 4b and share estimates should be interpreted as approximate indicators of spatial patterns rather than precise county-specific estimates. Fourth, LISA entails many simultaneous local tests, and results may be sensitive to spatial weights, county boundaries, and other areal definitions, commonly referred to as the modifiable areal unit problem (25). We therefore interpret LISA clusters as hypothesis-generating indicators of place-based inequities rather than definitive causal clusters. Fifth, urbanization-stratified time series were available through 2020, and this difference in data availability should be considered when comparing trends across strata. Finally, the study period included the COVID-19 pandemic years. Although the post-2015 increase in Stage 4b mortality began before the pandemic, mortality during 2020–2023 may reflect both underlying CKM progression and pandemic-related factors, including acute COVID-19 complications, delayed care, disruption of chronic disease management, and health-system strain. Because individual-level infection status, care disruption, and clinical trajectories were unavailable, we could not distinguish true cardiometabolic progression from acute pandemic-related contributions.

These findings have several implications. Advanced CKM mortality should be monitored not only as an absolute cardiovascular burden (Stage 4 AAMR) but also as a cardio–renal overlap phenotype captured by kidney failure involvement (Stage 4b; assessed as the Stage 4b share within Stage 4). The marked geographic decoupling between Stage 4 hotspots (Deep South/Appalachia) and county clusters of Stage 4b share (Great Plains–Mountain West corridor) implies that local prevention priorities may differ depending on whether the goal is to reduce overall advanced cardiovascular burden or to address disproportionate kidney failure involvement within that burden. From a kidney-health perspective, rising Stage 4b mortality after 2015 and clustered county patterns underscore the need to strengthen CKD detection and cardiometabolic risk control upstream, and to improve coordination of cardio–renal care pathways in high-share areas (e.g., timely nephrology referral, integrated management of diabetes/hypertension/heart failure, and access to evidence-based therapies). Future work should connect these descriptive patterns to contextual determinants (e.g., socioeconomic conditions, rurality and access measures, dialysis and transplantation resources, clinician availability) using multilevel and spatiotemporal models to identify modifiable levers for place-based implementation strategies. Additional analyses could evaluate robustness to alternative kidney failure definitions and more granular stratification (e.g., refined age bands within the non-elderly group and more detailed race/ethnicity categories) to identify populations and places where improvements have stalled.

## 5 Conclusions

From 1999 to 2023, AAMR for CKM Stage 4 and Stage 4b declined overall in the United States; however, trends shifted in the early 2010s, with Stage 4 increasing after 2013 and Stage 4b increasing after 2015. Geographic inequities were substantial and scale-dependent: Stage 4 burden clustered in the Deep South and Appalachia, whereas the proportional contribution of Stage 4b within Stage 4 exhibited a distinct Great Plains–Mountain West corridor and significant local clustering. These findings underscore the importance of monitoring both absolute burden and compositional measures of advanced CKM mortality and implementing place-based, interdisciplinary strategies to reduce inequities.

## Data Availability

The original contributions presented in the study are included in the article/Supplementary Material, further inquiries can be directed to the corresponding author.
