# Supplementary material for: National trends and county-level geographic disparities in mortality from operationally defined cardiovascular–kidney–metabolic stages 4 and 4b in the United States
Source: Front Cardiovasc Med. 2026 May 29;13:1838375. doi: 10.3389/fcvm.2026.1838375 (PMC13259836; doi:10.3389/fcvm.2026.1838375)
Supplement: Supplementary file 1 [file Datasheet1.docx]

**National Trends and County-Level Geographic Disparities in Mortality From Operationally Defined Cardiovascular–Kidney–Metabolic Stages 4 and 4b in the United States**

**Content**

[Supplementary Tables 4](#_Toc228731178)

[**Supplementary Table 1. AAMR and death counts for Stage 4 and Stage 4b CKM syndrome.** 4](#_Toc228731179)

[**Supplementary Table 2. Segmented Trends and APCs in AAMR for Stage 4 and Stage 4b CKM Syndrome in 1999–2023.** 6](#_Toc228731180)

[**Supplementary Table 3. Change in AAMR ratio and absolute AAMR difference between Stage 4 and Stage 4b CKM syndrome.** 10](#_Toc228731181)

[**Supplementary Table 4. Segmented temporal trends and APC in the AAMR and absolute rate difference of Stage 4/4b CKM syndrome.** 12](#_Toc228731182)

[**Supplementary Table 5. State-level mortality burden and long-term trends (AAPC) for Stage 4 CKM syndrome in 1999–2023.** 16](#_Toc228731183)

[**Supplementary Table 6. State-level mortality burden and long-term trends for Stage 4b CKM syndrome in 1999–2023.** 19](#_Toc228731184)

[**Supplementary Table 7. Segmented temporal trends and APC in mortality rates for Stage 4 CKM syndrome by state, 1999–2023.** 22](#_Toc228731185)

[**Supplementary Table 8. Segmented temporal trends and APC in mortality rates for Stage 4b CKM syndrome by state, 1999–2023.** 28](#_Toc228731186)

[**Supplementary Table 9. Sensitivity analyses for county-level suppressed death counts.** 36](#_Toc228731187)

[Supplementary Figures 37](#_Toc228731188)

[**Supplementary Figure 1. Temporal trends in AAMR for Stage 4 (A) and Stage 4b (B) CKM syndrome by sex, 1999–2023.** 37](#_Toc228731189)

[**Supplementary Figure 2. Temporal trends in AAMR for Stage 4 (A) and Stage 4b (B) CKM syndrome by age, 1999–2023.** 37](#_Toc228731190)

[**Supplementary Figure 3. Temporal trends in AAMR for Stage 4 (A) and Stage 4b (B) CKM syndrome by race, 1999–2023.** 38](#_Toc228731191)

[**Supplementary Figure 4. Temporal trends in AAMR for Stage 4 (A) and Stage 4b (B) CKM syndrome by census regions, 1999–2023.** 39](#_Toc228731192)

[**Supplementary Figure 5. Temporal trends in AAMR for Stage 4 (A) and Stage 4b (B) CKM syndrome by urbanization, 1999–2020.** 39](#_Toc228731193)

[**Supplementary Figure 6. Temporal trends in the AAMR ratio and absolute rate difference for CKM syndrome by sex, 1999–2023.** 40](#_Toc228731194)

[**Supplementary Figure 7. Temporal trends in the AAMR ratio and absolute rate difference for CKM syndrome by age, 1999–2023.** 40](#_Toc228731195)

[**Supplementary Figure 8. Temporal trends in the AAMR ratio and absolute rate difference for CKM syndrome by race, 1999–2023.** 41](#_Toc228731196)

[**Supplementary Figure 9. Temporal trends in the AAMR ratio and absolute rate difference for CKM syndrome by census regions, 1999–2023.** 41](#_Toc228731197)

[**Supplementary Figure 10. Temporal trends in the AAMR ratio and absolute rate difference for CKM syndrome by urbanization, 1999–2020.** 42](#_Toc228731198)

[**Supplementary Figure 11. State-level distribution of AAMR for CKM Stage 4, 2023.** 42](#_Toc228731199)

[**Supplementary Figure 12. State-level distribution of AAMR for CKM Stage 4b, 2023.** 43](#_Toc228731200)

[**Supplementary Figure 13. State-level variation in the proportional contribution (share) of CKM Stage 4b within Stage 4 (4b/4), 2023.** 43](#_Toc228731201)

[**Supplementary Figure 14. State-level distribution of the absolute AAMR gap between CKM Stage 4 and Stage 4b (Stage 4 − Stage 4b), 2023.** 44](#_Toc228731202)

[**Supplementary Figure 15. County-level geographic distribution (deciles) of age-adjusted mortality rates for CKM Stage 4 (A) and Stage 4b (B), 2023.** 45](#_Toc228731203)

[**Supplementary Figure 16. County-level geographic distribution (deciles) of the absolute AAMR gap between CKM Stage 4 and Stage 4b (Stage 4 − Stage 4b), 2023.** 46](#_Toc228731204)

[**Supplementary Figure 17. LISA cluster map for the Stage 4b share within CKM Stage 4 (4b/4), 2023.** 46](#_Toc228731205)

[**Supplementary Figure 18. County-level LISA cluster maps for CKM age-adjusted mortality rates for Stage 4 (A), Stage 4b (B), and the Stage 4b share within Stage 4 (4b/4) (C), 2023.** 47](#_Toc228731206)

# **Supplementary Tables**

## **Supplementary Table 1. AAMR and death counts for Stage 4 and Stage 4b CKM syndrome.**

|  | **1999 count** | **1999 AAMR, per 100000** | **2023 count** | **2023 AAMR, per 100000** |
| --- | --- | --- | --- | --- |
| **Stage 4** |  |  |  |  |
| **Overall** | 1080058 | 505.54 (504.59, 506.50) | 1169990 | 353.30 (352.66, 353.95) |
| **Sex** |  |  |  |  |
| Female | 573976 | 426.17 (425.07, 427.28) | 548377 | 288.62 (287.85, 289.39) |
| Male | 506082 | 615.30 (613.56, 617.03) | 621613 | 432.28 (431.18, 433.38) |
| **Age** |  |  |  |  |
| Elderly | 933908 | 2717.76 (2712.25, 2723.27) | 992266 | 1840.52 (1836.88, 1844.16) |
| Non-elderly | 146150 | 81.23 (80.81, 81.64) | 177724 | 68.05 (67.72, 68.37) |
| **Race** |  |  |  |  |
| Black | 109797 | 605.75 (602.13, 609.38) | 135887 | 402.48 (400.28, 404.68) |
| Other | 18176 | 331.32 (326.25, 336.39) | 47380 | 191.37 (189.63, 193.11) |
| White | 952085 | 499.25 (498.25, 500.26) | 986723 | 359.55 (358.84, 360.27) |
| **Urbanization**^a^ |  |  |  |  |
| Metro | 857586 | 496.79 (495.74, 497.84) | 995373 | 364.73 (364.01, 365.46) |
| Nonmetro | 222472 | 543.56 (541.29, 545.82) | 237454 | 446.96 (445.12, 448.79) |
| **Census regions** |  |  |  |  |
| Midwest | 270085 | 523.33 (521.35, 525.30) | 263522 | 377.63 (376.17, 379.09) |
| Northeast | 226656 | 496.60 (494.55, 498.64) | 189696 | 305.34 (303.95, 306.73) |
| South | 385433 | 516.99 (515.36, 518.62) | 468879 | 374.52 (373.44, 375.61) |
| West | 197884 | 472.14 (470.06, 474.23) | 247893 | 333.33 (332.00, 334.65) |
| **Stage 4b** |  |  |  |  |
| **Overall** | 98311 | 45.95 (45.66, 46.24) | 112838 | 33.98 (33.78, 34.18) |
| **Sex** |  |  |  |  |
| Female | 47601 | 36.23 (35.91, 36.56) | 50704 | 26.70 (26.47, 26.94) |
| Male | 50710 | 62.32 (61.77, 62.87) | 62134 | 43.66 (43.31, 44.01) |
| **Age** |  |  |  |  |
| Elderly | 85198 | 247.43 (245.76, 249.09) | 97889 | 181.38 (180.23, 182.52) |
| Non-elderly | 13113 | 7.30 (7.18, 7.43) | 14949 | 5.71 (5.61, 5.80) |
| **Race** |  |  |  |  |
| Black | 13884 | 76.03 (74.75, 77.31) | 15649 | 46.42 (45.67, 47.17) |
| Other | 2523 | 45.67 (43.80, 47.54) | 5094 | 20.48 (19.91, 21.05) |
| White | 81904 | 42.89 (42.60, 43.18) | 92095 | 33.41 (33.19, 33.63) |
| **Urbanization** |  |  |  |  |
| Metro | 77866 | 44.98 (44.67, 45.30) | 82560 | 30.26 (30.05, 30.46) |
| Nonmetro | 20445 | 49.85 (49.16, 50.53) | 20898 | 39.00 (38.47, 39.54) |
| **Census regions** |  |  |  |  |
| Midwest | 25533 | 49.55 (48.94, 50.15) | 25721 | 36.77 (36.32, 37.23) |
| Northeast | 20346 | 44.58 (43.97, 45.20) | 18006 | 28.92 (28.49, 29.35) |
| South | 33788 | 45.08 (44.60, 45.56) | 44274 | 35.19 (34.86, 35.52) |
| West | 18644 | 44.24 (43.60, 44.87) | 24837 | 33.27 (32.86, 33.69) |

ᵃ AAPCs for Urbanization strata were estimated for 1999–2020. AAMR: age-adjusted mortality rate, CKM: cardiovascular-kidney-metabolic.

## **Supplementary Table 2. Segmented Trends and APCs in AAMR for Stage 4 and Stage 4b CKM Syndrome in 1999–2023.**

| **Group** | **Segment** | **APC (95% CI)** | **P** |
| --- | --- | --- | --- |
| **Stage 4** |  |  |  |
| Overall | 1999-2013 | -3.05 (-3.54 to -2.64) | <0.001 |
| Overall | 2013-2023 | 1.17 (0.51 to 2.04) | 0.002 |
| Female | 1999-2013 | -3.26 (-3.71 to -2.89) | <0.001 |
| Female | 2013-2023 | 0.95 (0.32 to 1.76) | 0.006 |
| Male | 1999-2014 | -2.87 (-3.41 to -2.26) | 0.024 |
| Male | 2014-2021 | 2.37 (-3.67 to 5.57) | 0.134 |
| Male | 2021-2023 | -3.53 (-7.41 to 1.46) | 0.196 |
| Elderly | 1999-2013 | -3.19 (-3.67 to -2.8) | <0.001 |
| Elderly | 2013-2023 | 0.97 (0.32 to 1.79) | 0.006 |
| Non-elderly | 1999-2015 | -1.95 (-2.4 to -1.53) | 0.008 |
| Non-elderly | 2015-2021 | 4.47 (3.05 to 8.5) | 0.027 |
| Non-elderly | 2021-2023 | -5.1 (-9.62 to 0.17) | 0.059 |
| Black | 1999-2015 | -3.32 (-3.8 to -2.9) | 0.006 |
| Black | 2015-2021 | 3.81 (2.23 to 7.81) | 0.030 |
| Black | 2021-2023 | -5.67 (-10.15 to -0.22) | 0.045 |
| Other | 1999-2018 | -2.8 (-3.19 to -2.47) | 0.002 |
| Other | 2018-2021 | 6.8 (3.55 to 8.74) | 0.008 |
| Other | 2021-2023 | -8.00 (-12.04 to -3.51) | 0.006 |
| White | 1999-2013 | -2.94 (-3.4 to -2.56) | <0.001 |
| White | 2013-2023 | 1.31 (0.68 to 2.12) | <0.001 |
| Metro | 1999-2011 | -3.35 (-4.03 to -2.99) | 0.004 |
| Metro | 2011-2018 | -0.60 (-2.74 to 0.42) | 0.166 |
| Metro | 2018-2020 | 5.2 (0.98 to 7.38) | 0.006 |
| Nonmetro | 1999-2011 | -2.53 (-3 to -2.28) | 0.001 |
| Nonmetro | 2011-2018 | -0.25 (-1.50 to 0.55) | 0.375 |
| Nonmetro | 2018-2020 | 5.11 (1.84 to 6.93) | <0.001 |
| Midwest | 1999-2012 | -3.00 (-3.53 to -2.56) | <0.001 |
| Midwest | 2012-2023 | 1.01 (0.45 to 1.73) | 0.002 |
| Northeast | 1999-2012 | -3.31 (-3.84 to -2.91) | <0.001 |
| Northeast | 2012-2023 | -0.20 (-0.75 to 0.54) | 0.500 |
| South | 1999-2013 | -3.08 (-3.67 to -2.61) | <0.001 |
| South | 2013-2023 | 1.52 (0.78 to 2.5) | 0.001 |
| West | 1999-2013 | -3.16 (-3.65 to -2.73) | <0.001 |
| West | 2013-2023 | 1.38 (0.73 to 2.2) | <0.001 |
| **Stage 4b** |  |  |  |
| Overall | 1999-2012 | -1.53 (-2.59 to 1.23) | 0.132 |
| Overall | 2012-2015 | -13.8 (-18.2 to -4.41) | 0.006 |
| Overall | 2015-2023 | 4.63 (2.08 to 12.14) | 0.006 |
| Female | 1999-2012 | -1.73 (-2.81 to 0.98) | 0.104 |
| Female | 2012-2015 | -14.41 (-19 to -4.72) | 0.006 |
| Female | 2015-2023 | 4.83 (2.13 to 12.68) | 0.007 |
| Male | 1999-2012 | -1.63 (-2.66 to 1.12) | 0.116 |
| Male | 2012-2015 | -13.45 (-17.66 to -4.52) | 0.006 |
| Male | 2015-2023 | 4.23 (1.8 to 10.38) | 0.007 |
| Elderly | 1999-2012 | -1.44 (-2.5 to 1.43) | 0.154 |
| Elderly | 2012-2015 | -13.68 (-18.26 to -4.21) | 0.009 |
| Elderly | 2015-2023 | 4.08 (1.4 to 13.11) | 0.014 |
| Non-elderly | 1999-2012 | -2.17 (-3.48 to 2.03) | 0.108 |
| Non-elderly | 2012-2015 | -14.57 (-19.67 to -4.08) | 0.030 |
| Non-elderly | 2015-2023 | 8.52 (5.3 to 14.89) | 0.014 |
| Black | 1999-2012 | -2.46 (-3.54 to -0.45) | 0.033 |
| Black | 2012-2015 | -16.12 (-20.51 to -6.66) | 0.003 |
| Black | 2015-2023 | 5.23 (2.68 to 9.84) | 0.002 |
| Other | 1999-2012 | -2.43 (-3.59 to -0.16) | 0.043 |
| Other | 2012-2015 | -18.52 (-23.02 to -8.51) | 0.001 |
| Other | 2015-2023 | 3.23 (0.72 to 8.64) | 0.015 |
| White | 1999-2012 | -1.39 (-2.45 to 2) | 0.164 |
| White | 2012-2015 | -13.25 (-17.67 to -3.89) | 0.009 |
| White | 2015-2023 | 4.7 (2.03 to 12.95) | 0.010 |
| Metro | 1999-2012 | -1.69 (-2.68 to 0.45) | 0.075 |
| Metro | 2012-2015 | -13.4 (-17.47 to -5.25) | 0.008 |
| Metro | 2015-2020 | 3.48 (-0.84 to 14.98) | 0.088 |
| Nonmetro | 1999-2012 | -0.83 (-1.75 to 0.68) | 0.254 |
| Nonmetro | 2012-2015 | -13.62 (-17.67 to -6.04) | 0.005 |
| Nonmetro | 2015-2020 | 3.95 (-0.23 to 16.37) | 0.061 |
| Midwest | 1999-2012 | -1.51 (-2.46 to 0.44) | 0.086 |
| Midwest | 2012-2015 | -13.29 (-17.3 to -4.9) | 0.002 |
| Midwest | 2015-2023 | 4.27 (1.89 to 9.22) | 0.002 |
| Northeast | 1999-2012 | -2.12 (-3 to -0.21) | 0.043 |
| Northeast | 2012-2015 | -13.36 (-17.4 to -5.05) | 0.004 |
| Northeast | 2015-2023 | 3.31 (0.99 to 9.92) | 0.011 |
| South | 1999-2012 | -1.45 (-2.53 to 1.69) | 0.158 |
| South | 2012-2015 | -13.69 (-18.17 to -4.15) | 0.008 |
| South | 2015-2023 | 5.25 (2.63 to 12.82) | 0.006 |
| West | 1999-2012 | -1.16 (-2.32 to 1.33) | 0.277 |
| West | 2012-2015 | -14.89 (-19.43 to -5.17) | 0.004 |
| West | 2015-2023 | 5.02 (2.33 to 10.59) | 0.003 |

AAMR: age-adjusted mortality rate, CKM: cardiovascular-kidney-metabolic, APC: annual percent change.

## **Supplementary Table 3. Change in AAMR ratio and absolute AAMR difference between Stage 4 and Stage 4b CKM syndrome.**

|  | **1999 (95% CI)** | | | **2023 (95% CI)** | **AAPC (95% CI)** |
| --- | --- | --- | --- | --- | --- |
| **AAMR ratio** |  | | |  |  |
| **Overall** | 11.00 (10.93, 11.07) | | | 10.40 (10.33, 10.46) | -0.13 (-0.74 to 0.34) |
| **Age** |  | | |  |  |
| Elderly | 10.98 (10.91, 11.06) | | | 10.15 (10.08, 10.21) | -0.18 (-0.76 to 0.27) |
| Non-elderly | 11.12 (10.93, 11.32) | | | 11.93 (11.72, 12.13) | 0.07 (-0.78 to 0.66) |
| **Census regions** | |  |  |  |  |
| Midwest | 10.56 (10.43, 10.70) | | | 10.27 (10.14, 10.40) | 0.09 (-0.55 to 0.57) |
| Northeast | 11.14 (10.98, 11.30) | | | 10.56 (10.40, 10.72) | -0.02 (-0.61 to 0.42) |
| South | 11.47 (11.34, 11.60) | | | 10.64 (10.54, 10.75) | -0.25 (-0.83 to 0.2) |
| West | 10.67 (10.51, 10.84) | | | 10.02 (9.89, 10.15) | -0.26 (-0.91 to 0.26) |
| **Sex** |  | | |  |  |
| Female | 11.76 (11.65, 11.87) | | | 10.81 (10.71, 10.91) | -0.21 (-0.8 to 0.25) |
| Male | 9.87 (9.78, 9.97) | | | 9.90 (9.82, 9.99) | 0.11 (-0.52 to 0.58) |
| **Race** |  | | |  |  |
| Black | 7.97 (7.83, 8.11) | | | 8.67 (8.52, 8.82) | 0.34 (-0.3 to 0.85) |
| Other | 7.25 (6.94, 7.58) | | | 9.34 (9.08, 9.62) | 0.89 (0.28 to 1.43) |
| White | 11.64 (11.56, 11.72) | | | 10.76 (10.69, 10.84) | -0.19 (-0.78 to 0.27) |
| **Urbanization**^a^ |  | | |  |  |
| Metro | 11.04 (10.96, 11.13) | | | 12.06 (11.97, 12.14) | 0.51 (-0.33 to 1.27) |
| Nonmetro | 10.90 (10.75, 11.06) | | | 11.46 (11.30, 11.63) | 0.48 (-0.42 to 1.19) |
| **Absolute AAMR difference** |  | | |  |  |
| **Overall** | 459.60 (458.60, 460.59) | | | 319.33 (318.65, 320.00) | -1.31 (-1.5 to -1.12) |
| **Age** |  | | |  |  |
| Elderly | 2470.33 (2464.58, 2476.09) | | | 1659.14 (1655.32, 1662.96) | -1.48 (-1.66 to -1.3) |
| Non-elderly | 73.93 (73.49, 74.36) | | | 62.34 (62.00, 62.68) | -0.7 (-1.02 to -0.33) |
|  | |  |  |  |  |
| Midwest | 473.78 (471.72, 475.85) | | | 340.86 (339.33, 342.38) | -1.15 (-1.37 to -0.94) |
| Northeast | 452.01 (449.88, 454.15) | | | 276.42 (274.97, 277.88) | -2.08 (-2.3 to -1.81) |
| South | 471.91 (470.20, 473.61) | | | 339.33 (338.20, 340.46) | -1.21 (-1.43 to -0.98) |
| West | 427.91 (425.73, 430.09) | | | 300.05 (298.67, 301.44) | -1.3 (-1.5 to -1.1) |
| **Sex** |  | | |  |  |
| Female | 389.94 (388.78, 391.10) | | | 261.92 (261.11, 262.72) | -1.53 (-1.72 to -1.35) |
| Male | 552.98 (551.16, 554.80) | | | 388.62 (387.47, 389.77) | -1.44 (-1.71 to -1.12) |
| **Race** |  | | |  |  |
| Black | 529.72 (525.87, 533.57) | | | 356.06 (353.73, 358.38) | -1.8 (-2.12 to -1.37) |
| Other | 285.65 (280.25, 291.06) | | | 170.89 (169.06, 172.72) | -2.1 (-2.47 to -1.44) |
| White | 456.36 (455.32, 457.41) | | | 326.14 (325.39, 326.89) | -1.2 (-1.39 to -1.01) |
| **Urbanization** |  | | |  |  |
| Metro | 451.81 (450.71, 452.91) | | | 334.48 (333.72, 335.23) | -1.59 (-1.86 to -1.43) |
| Nonmetro | 493.71 (491.35, 496.07) | | | 407.95 (406.04, 409.86) | -1.02 (-1.28 to -0.87) |

ᵃ AAPCs for Urbanization strata were estimated for 1999–2020. AAPC, average annual percent change; CI, confidence interval.

## **Supplementary Table 4. Segmented temporal trends and APC in the AAMR and absolute rate difference of Stage 4/4b CKM syndrome.**

|  | **Group** | **Segment** | **APC (95% CI)** | **P** |
| --- | --- | --- | --- | --- |
| **Ratio** |  |  |  |  |
|  | Elderly | 1999-2012 | -1.8 (-3.23 to -0.87) | <0.001 |
|  | Elderly | 2012-2015 | 15.15 (5.48 to 19.52) | <0.001 |
|  | Elderly | 2015-2023 | -2.85 (-5.9 to -1.04) | 0.002 |
|  | Non-elderly | 1999-2012 | -0.06 (-3.29 to 1.20) | 0.753 |
|  | Non-elderly | 2012-2015 | 17.95 (4.61 to 23.88) | 0.001 |
|  | Non-elderly | 2015-2023 | -5.71 (-9.64 to -3.32) | 0.001 |
|  | US | 1999-2012 | -1.57 (-3.15 to -0.59) | 0.002 |
|  | US | 2012-2015 | 15.54 (5.40 to 20.16) | <0.001 |
|  | US | 2015-2023 | -3.18 (-6.37 to -1.34) | 0.001 |
|  | Midwest | 1999-2012 | -1.37 (-2.99 to -0.41) | 0.008 |
|  | Midwest | 2012-2015 | 15.31 (5.20 to 19.98) | 0.002 |
|  | Midwest | 2015-2023 | -2.78 (-6.31 to -0.81) | 0.008 |
|  | Northeast | 1999-2012 | -1.14 (-2.60 to -0.29) | 0.010 |
|  | Northeast | 2012-2015 | 14.58 (5.22 to 18.77) | <0.001 |
|  | Northeast | 2015-2023 | -3.25 (-6.25 to -1.47) | <0.001 |
|  | South | 1999-2012 | -1.68 (-3.24 to -0.69) | 0.001 |
|  | South | 2012-2015 | 15.51 (5.65 to 20.02) | <0.001 |
|  | South | 2015-2023 | -3.36 (-6.17 to -1.6) | <0.001 |
|  | West | 1999-2012 | -2.00 (-3.72 to -0.92) | <0.001 |
|  | West | 2012-2015 | 16.76 (5.78 to 21.78) | <0.001 |
|  | West | 2015-2023 | -3.25 (-6.85 to -1.26) | 0.002 |
|  | Female | 1999-2012 | -1.57 (-3.06 to -0.65) | 0.001 |
|  | Female | 2012-2015 | 16.00 (5.85 to 20.55) | <0.001 |
|  | Female | 2015-2023 | -3.55 (-6.65 to -1.68) | <0.001 |
|  | Male | 1999-2012 | -1.37 (-3.06 to -0.35) | 0.008 |
|  | Male | 2012-2015 | 15.21 (5.21 to 19.8) | 0.002 |
|  | Male | 2015-2023 | -2.72 (-5.8 to -0.9) | 0.007 |
|  | Black | 1999-2012 | -1.11 (-2.78 to -0.06) | 0.037 |
|  | Black | 2012-2015 | 18.18 (6.69 to 23.48) | <0.001 |
|  | Black | 2015-2023 | -3.38 (-6.68 to -1.33) | 0.001 |
|  | Other | 1999-2012 | -0.95 (-2.65 to 0.18) | 0.096 |
|  | Other | 2012-2015 | 20.62 (9.24 to 25.73) | <0.001 |
|  | Other | 2015-2023 | -2.77 (-5.46 to -0.89) | 0.006 |
|  | White | 1999-2012 | -1.61 (-3.12 to -0.66) | 0.001 |
|  | White | 2012-2015 | 15.02 (5.28 to 19.43) | 0.001 |
|  | White | 2015-2023 | -3.12 (-6.16 to -1.31) | 0.002 |
|  | Metro | 1999-2012 | -1.54 (-3.27 to -0.28) | 0.039 |
|  | Metro | 2012-2015 | 14.63 (0.07 to 20.06) | 0.050 |
|  | Metro | 2015-2020 | -2 (-11.07 to 3.34) | 0.238 |
|  | Nonmetro | 1999-2012 | -1.61 (-3.33 to -0.43) | 0.030 |
|  | Nonmetro | 2012-2015 | 15.53 (3.5 to 20.73) | 0.040 |
|  | Nonmetro | 2015-2020 | -2.41 (-11.36 to 2.58) | 0.162 |
| **Difference** |  |  |  |  |
|  | Elderly | 1999-2012 | -3.42 (-3.92 to -3.02) | <0.001 |
|  | Elderly | 2012-2023 | 0.86 (0.34 to 1.47) | 0.003 |
|  | Non-elderly | 1999-2012 | -2.37 (-3.08 to -1.68) | 0.017 |
|  | Non-elderly | 2012-2021 | 2.52 (-2.88 to 5.16) | 0.093 |
|  | Non-elderly | 2021-2023 | -3.92 (-7.82 to 1.75) | 0.210 |
|  | US | 1999-2012 | -3.25 (-3.76 to -2.83) | <0.001 |
|  | US | 2012-2023 | 1.04 (0.49 to 1.67) | <0.001 |
|  | Midwest | 1999-2011 | -3.23 (-3.88 to -2.71) | <0.001 |
|  | Midwest | 2011-2023 | 0.97 (0.44 to 1.61) | 0.001 |
|  | Northeast | 1999-2012 | -3.39 (-3.82 to -2.96) | 0.002 |
|  | Northeast | 2012-2021 | 0.38 (-3.59 to 2.19) | 0.166 |
|  | Northeast | 2021-2023 | -4.42 (-7.12 to -0.17) | 0.030 |
|  | South | 1999-2012 | -3.3 (-3.93 to -2.81) | <0.001 |
|  | South | 2012-2023 | 1.33 (0.72 to 2.09) | <0.001 |
|  | West | 1999-2012 | -3.4 (-3.93 to -2.96) | <0.001 |
|  | West | 2012-2023 | 1.24 (0.69 to 1.88) | <0.001 |
|  | Female | 1999-2012 | -3.45 (-3.94 to -3.06) | <0.001 |
|  | Female | 2012-2023 | 0.78 (0.26 to 1.39) | 0.006 |
|  | Male | 1999-2012 | -3.25 (-3.97 to -2.52) | 0.015 |
|  | Male | 2012-2021 | 1.64 (-4.24 to 4.06) | 0.142 |
|  | Male | 2021-2023 | -3.25 (-6.56 to 1.16) | 0.231 |
|  | Black | 1999-2012 | -3.88 (-4.71 to -2.94) | 0.011 |
|  | Black | 2012-2021 | 1.95 (-5.00 to 5.02) | 0.116 |
|  | Black | 2021-2023 | -4.64 (-8.48 to 1.23) | 0.167 |
|  | Other | 1999-2012 | -3.63 (-5.19 to -0.41) | 0.046 |
|  | Other | 2012-2021 | 0.86 (-5.95 to 5.19) | 0.180 |
|  | Other | 2021-2023 | -5.12 (-9.61 to 0.26) | 0.094 |
|  | White | 1999-2012 | -3.16 (-3.66 to -2.76) | <0.001 |
|  | White | 2012-2023 | 1.16 (0.64 to 1.78) | <0.001 |
|  | Metro | 1999-2011 | -3.48 (-4.12 to -3.09) | 0.002 |
|  | Metro | 2011-2018 | -0.02 (-3.5 to 0.82) | 0.665 |
|  | Metro | 2018-2020 | 4.57 (0.81 to 6.69) | <0.001 |
|  | Nonmetro | 1999-2011 | -2.69 (-3.42 to -2.13) | 0.009 |
|  | Nonmetro | 2011-2018 | 0.40 (-3.12 to 1.12) | 0.793 |
|  | Nonmetro | 2018-2020 | 4.24 (0.92 to 6.31) | <0.001 |

APC, annual percent change; CI, confidence interval.

## **Supplementary Table 5. State-level mortality burden and long-term trends (AAPC) for Stage 4 CKM syndrome in 1999–2023.**

| **State** | **1999 count** | **1999 AAMR (95% CI)** | **2023 count** | **2023 AAMR (95% CI)** | **AAPC (95% CI)** |
| --- | --- | --- | --- | --- | --- |
| Alabama | 18441 | 530.91(523.23, 538.58) | 17790 | 349.19(343.97, 354.41) | -1.87 (-2.22 to -1.67) |
| Alaska | 910 | 440.69(409.65, 471.73) | 1755 | 330.16(313.92, 346.40) | -1.02 (-1.62 to -0.34) |
| Arizona | 15729 | 414.44(407.93, 420.95) | 24593 | 309.13(305.22, 313.05) | -1.16 (-1.62 to -0.72) |
| Arkansas | 12362 | 543.37(533.78, 552.96) | 14008 | 458.17(450.47, 465.86) | -0.8 (-1.1 to -0.53) |
| California | 110291 | 503.38(500.41, 506.36) | 118074 | 323.97(322.11, 325.83) | -1.66 (-1.87 to -1.44) |
| Colorado | 10694 | 420.59(412.58, 428.59) | 17293 | 346.71(341.48, 351.95) | -0.9 (-1.17 to -0.6) |
| Connecticut | 13302 | 441.46(433.95, 448.98) | 10623 | 264.99(259.90, 270.08) | -2.18 (-2.45 to -1.96) |
| Delaware | 3069 | 524.66(506.02, 543.31) | 4540 | 402.93(390.94, 414.92) | -1.04 (-1.36 to -0.77) |
| District of Columbia | 2115 | 483.32(462.72, 503.93) | 1653 | 307.63(292.71, 322.55) | -2.15 (-2.71 to -1.74) |
| Florida | 72115 | 439.42(436.19, 442.65) | 94362 | 330.71(328.57, 332.86) | -0.99 (-1.2 to -0.78) |
| Georgia | 23762 | 491.31(485.02, 497.59) | 30187 | 324.34(320.62, 328.06) | -1.71 (-2.03 to -1.4) |
| Hawaii | 3537 | 377.56(365.09, 390.03) | 4611 | 259.29(251.60, 266.97) | -1.47 (-1.69 to -1.25) |
| Idaho | 4158 | 462.31(448.26, 476.37) | 6363 | 357.31(348.40, 366.22) | -1.07 (-1.36 to -0.75) |
| Illinois | 49889 | 531.10(526.44, 535.76) | 39878 | 318.42(315.27, 321.57) | -1.86 (-2.09 to -1.62) |
| Indiana | 25047 | 541.21(534.50, 547.91) | 26766 | 409.85(404.89, 414.82) | -0.99 (-1.23 to -0.74) |
| Iowa | 13724 | 477.81(469.76, 485.87) | 13022 | 378.69(372.10, 385.28) | -0.76 (-0.97 to -0.56) |
| Kansas | 10881 | 468.45(459.61, 477.30) | 10446 | 361.21(354.20, 368.21) | -1.17 (-1.46 to -0.91) |
| Kentucky | 17674 | 579.72(571.16, 588.28) | 20070 | 457.07(450.64, 463.50) | -0.94 (-1.31 to -0.42) |
| Louisiana | 16664 | 536.16(528.00, 544.31) | 17058 | 395.64(389.61, 401.68) | -0.97 (-1.25 to -0.68) |
| Maine | 5520 | 490.27(477.33, 503.21) | 5718 | 333.31(324.53, 342.09) | -1.6 (-1.78 to -1.42) |
| Maryland | 19485 | 536.97(529.41, 544.54) | 22002 | 363.08(358.24, 367.92) | -1.73 (-1.94 to -1.54) |
| Massachusetts | 23597 | 431.08(425.57, 436.59) | 20911 | 285.50(281.60, 289.41) | -1.73 (-1.89 to -1.57) |
| Michigan | 41610 | 561.31(555.91, 566.71) | 41977 | 404.53(400.60, 408.46) | -1.13 (-1.38 to -0.89) |
| Minnesota | 16543 | 423.79(417.31, 430.27) | 20912 | 360.43(355.49, 365.36) | -0.44 (-0.68 to -0.19) |
| Mississippi | 12516 | 593.62(583.21, 604.03) | 13483 | 474.09(465.95, 482.23) | -0.96 (-1.41 to -0.68) |
| Missouri | 26025 | 552.68(545.96, 559.40) | 24489 | 386.95(382.04, 391.86) | -1.33 (-1.53 to -1.13) |
| Montana | 3209 | 427.56(412.76, 442.35) | 4258 | 352.88(342.09, 363.68) | -0.8 (-1.17 to -0.47) |
| Nebraska | 6783 | 448.61(437.88, 459.35) | 7554 | 394.91(385.92, 403.89) | -0.32 (-0.6 to -0.02) |
| Nevada | 5476 | 458.49(445.93, 471.04) | 10479 | 354.57(347.64, 361.49) | -0.96 (-1.29 to -0.56) |
| New Hampshire | 4294 | 471.00(456.91, 485.10) | 5397 | 343.82(334.50, 353.14) | -1.43 (-1.69 to -1.2) |
| New Jersey | 34255 | 501.97(496.65, 507.28) | 26409 | 276.88(273.52, 280.25) | -2.31 (-2.59 to -2.06) |
| New Mexico | 5384 | 434.34(422.70, 445.97) | 8104 | 364.14(356.05, 372.23) | -0.73 (-0.94 to -0.53) |
| New York | 76509 | 505.68(502.10, 509.26) | 61426 | 289.22(286.91, 291.53) | -2.33 (-2.6 to -2.16) |
| North Carolina | 30958 | 538.48(532.47, 544.50) | 39917 | 385.18(381.35, 389.02) | -1.29 (-1.52 to -1.08) |
| North Dakota | 3057 | 489.12(471.58, 506.66) | 2802 | 358.05(344.54, 371.57) | -1.22 (-1.49 to -1.03) |
| Ohio | 51899 | 568.11(563.22, 573.01) | 47941 | 395.33(391.75, 398.92) | -1.52 (-1.81 to -1.29) |
| Oklahoma | 16782 | 599.21(590.14, 608.28) | 17581 | 470.47(463.44, 477.50) | -1.06 (-1.41 to -0.73) |
| Oregon | 12774 | 459.80(451.83, 467.78) | 17700 | 404.99(398.95, 411.03) | -0.45 (-0.66 to -0.21) |
| Pennsylvania | 61973 | 529.28(525.10, 533.46) | 51943 | 353.01(349.94, 356.08) | -1.54 (-1.74 to -1.33) |
| Rhode Island | 4898 | 505.23(491.02, 519.44) | 4423 | 363.84(353.00, 374.68) | -1.43 (-1.71 to -1.17) |
| South Carolina | 15317 | 538.26(529.68, 546.83) | 21625 | 397.30(391.88, 402.71) | -1.17 (-1.41 to -0.92) |
| South Dakota | 3226 | 458.66(442.70, 474.62) | 3573 | 385.76(372.88, 398.64) | -0.66 (-0.96 to -0.35) |
| Tennessee | 24194 | 567.92(560.75, 575.08) | 29667 | 440.43(435.33, 445.53) | -1.09 (-1.44 to -0.8) |
| Texas | 67020 | 534.85(530.79, 538.91) | 85689 | 367.47(364.97, 369.96) | -1.36 (-1.63 to -1.1) |
| Utah | 4654 | 406.48(394.77, 418.19) | 7437 | 338.88(331.06, 346.70) | -0.7 (-1.01 to -0.37) |
| Vermont | 2308 | 477.13(457.65, 496.61) | 2846 | 381.83(367.54, 396.11) | -0.94 (-1.23 to -0.64) |
| Virginia | 22529 | 473.19(466.99, 479.40) | 29864 | 358.45(354.34, 362.55) | -1.09 (-1.36 to -0.81) |
| Washington | 19430 | 464.63(458.09, 471.17) | 25114 | 349.21(344.84, 353.58) | -1.04 (-1.27 to -0.81) |
| West Virginia | 10430 | 631.63(619.50, 643.77) | 9383 | 459.47(449.97, 468.96) | -1.03 (-1.3 to -0.75) |
| Wisconsin | 21401 | 479.01(472.58, 485.43) | 24162 | 394.80(389.76, 399.85) | -0.51 (-0.78 to -0.24) |
| Wyoming | 1638 | 472.45(449.52, 495.37) | 2112 | 361.73(346.03, 377.43) | -1 (-1.36 to -0.6) |

AAMR: age-adjusted mortality rates, AAPC, average annual percent change; CI, confidence interval.

## **Supplementary Table 6. State-level mortality burden and long-term trends for Stage 4b CKM syndrome in 1999–2023.**

| **State** | **1999 count** | **1999 AAMR (95% CI)** | **2023 count** | **2023 AAMR (95% CI)** | **AAPC (95% CI)** |
| --- | --- | --- | --- | --- | --- |
| Alabama | 1780 | 51.00 (48.63, 53.38) | 1621 | 31.47 (29.91, 33.02) | -2.09 (-2.85 to -1.14) |
| Alaska | 88 | 43.34 (34.15, 54.24) | 154 | 30.46 (25.40, 35.52) | -1.53 (-2.79 to -0.02) |
| Arizona | 1036 | 26.90 (25.26, 28.55) | 1863 | 23.31 (22.24, 24.39) | -0.64 (-1.97 to 0.91) |
| Arkansas | 919 | 40.39 (37.77, 43.00) | 1286 | 41.80 (39.48, 44.11) | -0.03 (-0.67 to 0.9) |
| California | 11780 | 53.48 (52.51, 54.44) | 12090 | 33.12 (32.52, 33.71) | -1.84 (-2.53 to -0.91) |
| Colorado | 823 | 32.60 (30.37, 34.84) | 2163 | 43.55 (41.69, 45.41) | 1.26 (0.36 to 2.3) |
| Connecticut | 1089 | 36.28 (34.12, 38.44) | 906 | 22.59 (21.10, 24.07) | -1.78 (-2.5 to -0.95) |
| Delaware | 363 | 61.12 (54.81, 67.44) | 378 | 33.27 (29.84, 36.71) | -2.48 (-3.85 to -1.56) |
| District of Columbia | 232 | 53.04 (46.22, 59.87) | 189 | 35.46 (30.37, 40.54) | -1.65 (-2.5 to -0.74) |
| Florida | 5440 | 33.09 (32.21, 33.98) | 7653 | 26.75 (26.14, 27.36) | -0.68 (-1.43 to 0.45) |
| Georgia | 1862 | 38.06 (36.32, 39.79) | 2630 | 28.07 (26.98, 29.16) | -1.26 (-1.89 to -0.38) |
| Hawaii | 490 | 51.87 (47.27, 56.47) | 398 | 22.68 (20.39, 24.97) | -3.05 (-3.63 to -2.4) |
| Idaho | 341 | 37.97 (33.94, 42.00) | 572 | 32.00 (29.34, 34.66) | -0.84 (-2.33 to 0.33) |
| Illinois | 4634 | 49.44 (48.02, 50.86) | 3622 | 28.88 (27.93, 29.83) | -2.29 (-2.83 to -1.58) |
| Indiana | 2426 | 52.35 (50.27, 54.44) | 2687 | 41.02 (39.46, 42.59) | -0.92 (-1.75 to -0.09) |
| Iowa | 1183 | 41.57 (39.18, 43.96) | 1353 | 39.12 (37.02, 41.23) | 0.24 (-0.29 to 0.97) |
| Kansas | 925 | 40.02 (37.43, 42.62) | 989 | 33.91 (31.78, 36.05) | -0.27 (-1.24 to 0.56) |
| Kentucky | 1470 | 48.16 (45.69, 50.62) | 2580 | 58.92 (56.61, 61.22) | 0.83 (0.17 to 2.01) |
| Louisiana | 1395 | 44.73 (42.37, 47.08) | 1153 | 26.64 (25.07, 28.20) | -1.75 (-2.34 to -0.99) |
| Maine | 556 | 49.48 (45.36, 53.59) | 462 | 27.43 (24.88, 29.98) | -2.35 (-3.04 to -1.59) |
| Maryland | 2183 | 59.63 (57.12, 62.14) | 2345 | 38.45 (36.88, 40.02) | -1.75 (-2.28 to -1.12) |
| Massachusetts | 2066 | 37.89 (36.25, 39.53) | 2377 | 32.34 (31.03, 33.65) | -0.93 (-1.31 to -0.44) |
| Michigan | 3864 | 51.92 (50.28, 53.56) | 3557 | 34.10 (32.97, 35.24) | -1.64 (-2.23 to -0.86) |
| Minnesota | 1599 | 41.20 (39.17, 43.22) | 2424 | 41.64 (39.97, 43.31) | 0.16 (-0.57 to 1.3) |
| Mississippi | 1131 | 53.56 (50.44, 56.68) | 1149 | 40.03 (37.68, 42.38) | -0.78 (-1.47 to 0.09) |
| Missouri | 2360 | 50.16 (48.14, 52.19) | 2410 | 38.05 (36.51, 39.58) | -1.41 (-1.92 to -0.78) |
| Montana | 261 | 34.82 (30.60, 39.05) | 384 | 31.94 (28.69, 35.19) | -0.69 (-1.54 to 0.37) |
| Nebraska | 574 | 38.44 (35.28, 41.60) | 936 | 48.54 (45.41, 51.68) | 0.87 (0.32 to 1.57) |
| Nevada | 418 | 34.20 (30.82, 37.59) | 843 | 28.29 (26.34, 30.24) | -1.12 (-2.04 to -0.09) |
| New Hampshire | 373 | 41.11 (36.94, 45.29) | 536 | 33.82 (30.93, 36.72) | -1.24 (-2.12 to 0.07) |
| New Jersey | 3208 | 46.90 (45.28, 48.53) | 2957 | 30.90 (29.78, 32.02) | -1.67 (-2.17 to -1.04) |
| New Mexico | 390 | 31.01 (27.92, 34.10) | 428 | 19.00 (17.16, 20.83) | -0.96 (-1.54 to -0.22) |
| New York | 5701 | 37.74 (36.76, 38.72) | 4193 | 19.74 (19.14, 20.35) | -2.59 (-3.17 to -1.89) |
| North Carolina | 3151 | 54.33 (52.43, 56.24) | 3936 | 37.92 (36.72, 39.12) | -1.35 (-1.9 to -0.63) |
| North Dakota | 339 | 53.70 (47.92, 59.48) | 339 | 42.58 (37.98, 47.17) | -0.4 (-1.11 to 0.48) |
| Ohio | 5492 | 59.88 (58.30, 61.46) | 4163 | 34.20 (33.15, 35.25) | -2.48 (-3.24 to -1.62) |
| Oklahoma | 1183 | 42.25 (39.84, 44.66) | 1592 | 42.85 (40.73, 44.98) | -0.18 (-0.82 to 0.75) |
| Oregon | 916 | 33.10 (30.96, 35.25) | 1980 | 45.16 (43.15, 47.17) | 1.29 (0.69 to 2.26) |
| Pennsylvania | 6694 | 56.95 (55.58, 58.32) | 6040 | 40.84 (39.80, 41.88) | -1.42 (-1.99 to -0.7) |
| Rhode Island | 470 | 48.58 (44.17, 52.99) | 308 | 25.49 (22.62, 28.37) | -2.89 (-3.92 to -1.99) |
| South Carolina | 1466 | 50.80 (48.19, 53.42) | 2214 | 40.10 (38.39, 41.81) | -1.12 (-1.62 to -0.33) |
| South Dakota | 265 | 37.53 (32.98, 42.08) | 446 | 48.62 (44.05, 53.19) | 1.05 (0.46 to 1.89) |
| Tennessee | 2076 | 48.49 (46.40, 50.58) | 2540 | 37.67 (36.18, 39.16) | -1.08 (-1.81 to 0.04) |
| Texas | 5986 | 47.48 (46.27, 48.69) | 9244 | 39.37 (38.56, 40.18) | -1.18 (-2.02 to -0.28) |
| Utah | 318 | 27.55 (24.52, 30.59) | 814 | 37.25 (34.65, 39.84) | 0.82 (0.11 to 1.5) |
| Vermont | 189 | 39.33 (33.71, 44.94) | 227 | 30.13 (26.16, 34.11) | -1.58 (-2.43 to -0.59) |
| Virginia | 2111 | 44.01 (42.12, 45.89) | 2924 | 35.07 (33.79, 36.35) | -0.73 (-1.65 to 0.63) |
| Washington | 1670 | 40.01 (38.09, 41.93) | 2937 | 40.63 (39.15, 42.12) | 0.14 (-0.64 to 1.33) |
| West Virginia | 1040 | 62.60 (58.79, 66.41) | 840 | 40.88 (38.07, 43.69) | -1.6 (-2.65 to -0.22) |
| Wisconsin | 1872 | 42.00 (40.10, 43.91) | 2795 | 45.41 (43.71, 47.11) | 0.3 (-0.6 to 1.1) |
| Wyoming | 113 | 32.38 (26.39, 38.36) | 211 | 36.12 (31.16, 41.09) | 0.05 (-0.68 to 0.72) |

AAMR: age-adjusted mortality rates, AAPC, average annual percent change; CI, confidence interval.

## **Supplementary Table 7. Segmented temporal trends and APC in mortality rates for Stage 4 CKM syndrome by state, 1999–2023.**

| **State** | **Segment** | **APC (95% CI)** | **P** |
| --- | --- | --- | --- |
| Alabama | 1999-2018 | -2.42 (-2.71 to -2.19) | 0.001 |
| Alabama | 2018-2021 | 6.54 (3.5 to 8.21) | 0.005 |
| Alabama | 2021-2023 | -8.54 (-12.86 to -3.93) | 0.004 |
| Alaska | 1999-2018 | -1.89 (-2.74 to -0.43) | 0.047 |
| Alaska | 2018-2021 | 9.56 (-5.66 to 12.74) | 0.146 |
| Alaska | 2021-2023 | -7.55 (-14.54 to 3.17) | 0.153 |
| Arizona | 1999-2010 | -3.64 (-5.67 to -2.5) | <0.001 |
| Arizona | 2010-2023 | 0.98 (0.17 to 2.29) | 0.022 |
| Arkansas | 1999-2015 | -1.94 (-2.34 to -1.57) | 0.014 |
| Arkansas | 2015-2021 | 3.48 (-0.76 to 6.72) | 0.053 |
| Arkansas | 2021-2023 | -4.11 (-7.98 to 1.31) | 0.090 |
| California | 1999-2014 | -3.29 (-3.73 to -2.92) | <0.001 |
| California | 2014-2023 | 1.12 (0.35 to 2.15) | 0.006 |
| Colorado | 1999-2012 | -3.44 (-4.05 to -2.85) | 0.013 |
| Colorado | 2012-2021 | 3.2 (-3.76 to 5.5) | 0.094 |
| Colorado | 2021-2023 | -2.26 (-5.59 to 2.49) | 0.360 |
| Connecticut | 1999-2012 | -3.64 (-4.08 to -3.28) | 0.006 |
| Connecticut | 2012-2021 | 0.73 (0.08 to 2.3) | 0.043 |
| Connecticut | 2021-2023 | -5.47 (-8.71 to -1.1) | 0.015 |
| Delaware | 1999-2013 | -3.29 (-4.8 to -2.76) | 0.015 |
| Delaware | 2013-2019 | 0.12 (-3.06 to 2.2) | 0.997 |
| Delaware | 2019-2023 | 5.4 (2.83 to 10.01) | 0.001 |
| District of Columbia | 1999-2018 | -3.05 (-3.58 to -2.65) | 0.010 |
| District of Columbia | 2018-2021 | 9.73 (-0.79 to 12.95) | 0.052 |
| District of Columbia | 2021-2023 | -10.08 (-16.82 to 0.14) | 0.052 |
| Florida | 1999-2013 | -3.29 (-3.82 to -2.86) | <0.001 |
| Florida | 2013-2023 | 2.32 (1.64 to 3.17) | <0.001 |
| Georgia | 1999-2011 | -4.01 (-5.18 to -3.26) | <0.001 |
| Georgia | 2011-2023 | 0.64 (-0.1 to 1.69) | 0.085 |
| Hawaii | 1999-2012 | -3.24 (-3.83 to -2.78) | <0.001 |
| Hawaii | 2012-2023 | 0.65 (0.07 to 1.41) | 0.027 |
| Idaho | 1999-2013 | -2.73 (-3.28 to -2.22) | 0.005 |
| Idaho | 2013-2021 | 2.84 (1.85 to 5.33) | 0.034 |
| Idaho | 2021-2023 | -4.7 (-8.14 to 0.61) | 0.084 |
| Illinois | 1999-2013 | -3.55 (-4.09 to -3.11) | <0.001 |
| Illinois | 2013-2023 | 0.56 (-0.22 to 1.61) | 0.141 |
| Indiana | 1999-2010 | -3.09 (-4.01 to -2.43) | <0.001 |
| Indiana | 2010-2023 | 0.83 (0.31 to 1.5) | 0.003 |
| Iowa | 1999-2011 | -2.62 (-3.23 to -2.15) | <0.001 |
| Iowa | 2011-2023 | 1.13 (0.64 to 1.75) | <0.001 |
| Kansas | 1999-2013 | -2.69 (-3.21 to -2.25) | 0.016 |
| Kansas | 2013-2021 | 2.19 (-2.67 to 4.52) | 0.087 |
| Kansas | 2021-2023 | -3.68 (-7.16 to 1.3) | 0.148 |
| Kentucky | 1999-2014 | -2.46 (-3.63 to -0.31) | 0.046 |
| Kentucky | 2014-2021 | 3 (-5.15 to 7.12) | 0.261 |
| Kentucky | 2021-2023 | -2.93 (-7.67 to 2.33) | 0.414 |
| Louisiana | 1999-2013 | -2.92 (-3.62 to -2.37) | <0.001 |
| Louisiana | 2013-2023 | 1.82 (0.91 to 3.08) | <0.001 |
| Maine | 1999-2011 | -3.66 (-4.2 to -3.23) | <0.001 |
| Maine | 2011-2023 | 0.51 (0.05 to 1.05) | 0.026 |
| Maryland | 1999-2015 | -3.69 (-3.95 to -3.45) | <0.001 |
| Maryland | 2015-2021 | 4.73 (3.83 to 6.73) | <0.001 |
| Maryland | 2021-2023 | -4.6 (-7.35 to -0.74) | 0.016 |
| Massachusetts | 1999-2012 | -3.35 (-3.79 to -3.01) | <0.001 |
| Massachusetts | 2012-2023 | 0.23 (-0.25 to 0.83) | 0.296 |
| Michigan | 1999-2013 | -2.7 (-3.3 to -2.24) | <0.001 |
| Michigan | 2013-2023 | 1.11 (0.34 to 2.23) | 0.008 |
| Minnesota | 1999-2010 | -3.15 (-4.04 to -2.46) | <0.001 |
| Minnesota | 2010-2023 | 1.91 (1.4 to 2.57) | <0.001 |
| Mississippi | 1999-2018 | -2.03 (-2.41 to -1.73) | 0.001 |
| Mississippi | 2018-2021 | 12.08 (7.82 to 14.55) | 0.004 |
| Mississippi | 2021-2023 | -8.78 (-14.28 to -2.86) | 0.012 |
| Missouri | 1999-2014 | -2.82 (-3.23 to -2.48) | <0.001 |
| Missouri | 2014-2023 | 1.21 (0.46 to 2.22) | 0.002 |
| Montana | 1999-2014 | -2.15 (-2.77 to -1.64) | 0.006 |
| Montana | 2014-2021 | 3.66 (2.35 to 7.47) | 0.028 |
| Montana | 2021-2023 | -5.74 (-10.43 to 0.06) | 0.053 |
| Nebraska | 1999-2013 | -1.82 (-2.61 to -1.27) | <0.001 |
| Nebraska | 2013-2023 | 1.82 (0.96 to 3.26) | <0.001 |
| Nevada | 1999-2011 | -4.51 (-5.78 to -3.53) | <0.001 |
| Nevada | 2011-2023 | 2.73 (1.93 to 3.85) | <0.001 |
| New Hampshire | 1999-2009 | -3.68 (-5.56 to -3.01) | 0.004 |
| New Hampshire | 2009-2016 | -1.19 (-3.1 to 0.37) | 0.115 |
| New Hampshire | 2016-2023 | 1.61 (0.58 to 4.28) | 0.014 |
| New Jersey | 1999-2009 | -3.71 (-5.12 to -3.03) | <0.001 |
| New Jersey | 2009-2023 | -1.3 (-1.76 to -0.53) | 0.012 |
| New Mexico | 1999-2015 | -2.26 (-2.56 to -1.97) | 0.007 |
| New Mexico | 2015-2021 | 3.95 (2.82 to 6.19) | 0.038 |
| New Mexico | 2021-2023 | -2.17 (-4.92 to 1.6) | 0.280 |
| New York | 1999-2017 | -3 (-3.25 to -2.8) | <0.001 |
| New York | 2017-2020 | 4.35 (1.43 to 5.85) | 0.005 |
| New York | 2020-2023 | -4.77 (-8.41 to -2.81) | 0.002 |
| North Carolina | 1999-2010 | -3.51 (-4.64 to -3.04) | 0.002 |
| North Carolina | 2010-2017 | -0.99 (-2.5 to 0.55) | 0.159 |
| North Carolina | 2017-2023 | 2.54 (1.5 to 4.77) | 0.003 |
| North Dakota | 1999-2016 | -2.12 (-2.45 to -1.83) | 0.011 |
| North Dakota | 2016-2020 | 3.5 (-2.03 to 6.31) | 0.078 |
| North Dakota | 2020-2023 | -2.24 (-6.25 to 0.91) | 0.088 |
| Ohio | 1999-2016 | -2.66 (-2.99 to -2.37) | 0.007 |
| Ohio | 2016-2021 | 3.79 (2.01 to 7.5) | 0.040 |
| Ohio | 2021-2023 | -4.69 (-8.49 to 0.08) | 0.053 |
| Oklahoma | 1999-2012 | -2.54 (-3.67 to -1.87) | <0.001 |
| Oklahoma | 2012-2023 | 0.71 (-0.16 to 2.13) | 0.093 |
| Oregon | 1999-2013 | -2.61 (-3.15 to -2.16) | <0.001 |
| Oregon | 2013-2023 | 2.67 (1.98 to 3.55) | <0.001 |
| Pennsylvania | 1999-2012 | -3.26 (-3.8 to -2.84) | <0.001 |
| Pennsylvania | 2012-2023 | 0.54 (-0.05 to 1.31) | 0.072 |
| Rhode Island | 1999-2013 | -2.84 (-3.59 to -2.34) | <0.001 |
| Rhode Island | 2013-2023 | 0.57 (-0.30 to 1.94) | 0.185 |
| South Carolina | 1999-2011 | -3.27 (-4.07 to -2.68) | <0.001 |
| South Carolina | 2011-2023 | 0.99 (0.44 to 1.72) | 0.003 |
| South Dakota | 1999-2014 | -2.06 (-2.55 to -1.62) | 0.009 |
| South Dakota | 2014-2021 | 3.4 (-1.02 to 6.54) | 0.058 |
| South Dakota | 2021-2023 | -3.95 (-7.8 to 1.81) | 0.154 |
| Tennessee | 1999-2017 | -2.14 (-2.53 to -1.78) | 0.021 |
| Tennessee | 2017-2021 | 5.52 (-2.25 to 9.1) | 0.083 |
| Tennessee | 2021-2023 | -4.35 (-8.84 to 2.26) | 0.104 |
| Texas | 1999-2012 | -3.26 (-4.03 to -2.69) | <0.001 |
| Texas | 2012-2023 | 0.93 (0.23 to 1.86) | 0.012 |
| Utah | 1999-2010 | -3.27 (-4.68 to -2.38) | <0.001 |
| Utah | 2010-2023 | 1.53 (0.91 to 2.42) | <0.001 |
| Vermont | 1999-2012 | -2.28 (-3.24 to -1.68) | <0.001 |
| Vermont | 2012-2023 | 0.68 (-0.06 to 1.93) | 0.068 |
| Virginia | 1999-2014 | -3.16 (-3.76 to -2.68) | <0.001 |
| Virginia | 2014-2023 | 2.45 (1.50 to 3.80) | <0.001 |
| Washington | 1999-2014 | -2.51 (-3.01 to -2.12) | <0.001 |
| Washington | 2014-2023 | 1.45 (0.68 to 2.53) | 0.001 |
| West Virginia | 1999-2014 | -2.57 (-3.11 to -2.14) | <0.001 |
| West Virginia | 2014-2023 | 1.57 (0.66 to 2.95) | 0.003 |
| Wisconsin | 1999-2012 | -2.95 (-3.68 to -2.36) | <0.001 |
| Wisconsin | 2012-2023 | 2.45 (1.69 to 3.4) | <0.001 |
| Wyoming | 1999-2013 | -2.89 (-3.68 to -2.12) | 0.023 |
| Wyoming | 2013-2021 | 3.14 (-3.95 to 6.81) | 0.123 |
| Wyoming | 2021-2023 | -3.78 (-8.31 to 2.22) | 0.258 |

APC, annual percent change; CKM: cardiovascular–kidney–metabolic.

## **Supplementary Table 8. Segmented temporal trends and APC in mortality rates for Stage 4b CKM syndrome by state, 1999–2023.**

| **State** | **Segment** | **APC (95% CI)** | **P** |
| --- | --- | --- | --- |
| Alabama | 1999-2012 | -1.92 (-3.15 to 0.29) | 0.073 |
| Alabama | 2012-2015 | -19.62 (-24.93 to -7.58) | 0.003 |
| Alabama | 2015-2023 | 5.14 (1.72 to 19.32) | 0.013 |
| Alaska | 1999-2023 | -1.53 (-2.79 to -0.02) | 0.048 |
| Arizona | 1999-2023 | -0.64 (-1.97 to 0.91) | 0.418 |
| Arkansas | 1999-2012 | 0.06 (-1.07 to 2.5) | 0.734 |
| Arkansas | 2012-2015 | -14.8 (-19.63 to -4.2) | 0.004 |
| Arkansas | 2015-2023 | 5.98 (3.11 to 15.98) | 0.004 |
| California | 1999-2012 | -1.63 (-2.88 to 0.85) | 0.135 |
| California | 2012-2015 | -17.34 (-22.48 to -6.63) | 0.002 |
| California | 2015-2023 | 4.33 (1.25 to 12.88) | 0.010 |
| Colorado | 1999-2014 | -2.38 (-5.55 to -0.57) | 0.012 |
| Colorado | 2014-2023 | 7.64 (4.49 to 15.41) | <0.001 |
| Connecticut | 1999-2003 | 3.72 (-1.8 to 16.31) | 0.208 |
| Connecticut | 2003-2015 | -4.96 (-11.93 to -3.91) | 0.006 |
| Connecticut | 2015-2023 | 0.4 (-2.06 to 7.48) | 0.691 |
| Delaware | 1999-2020 | -4.11 (-7.27 to -3.22) | 0.023 |
| Delaware | 2020-2023 | 9.75 (-3.05 to 28.35) | 0.210 |
| District of Columbia | 1999-2012 | -2.44 (-3.81 to -0.39) | 0.028 |
| District of Columbia | 2012-2015 | -23.94 (-29.98 to -11.28) | <0.001 |
| District of Columbia | 2015-2023 | 9.72 (5.59 to 17.46) | <0.001 |
| Florida | 1999-2012 | -2.14 (-3.79 to 6.64) | 0.140 |
| Florida | 2012-2015 | -11.89 (-17.14 to 6.37) | 0.071 |
| Florida | 2015-2023 | 6.41 (1.75 to 17.32) | 0.046 |
| Georgia | 1999-2012 | -1.6 (-2.77 to 0.76) | 0.122 |
| Georgia | 2012-2015 | -17.72 (-22.65 to -6.78) | 0.002 |
| Georgia | 2015-2023 | 6.31 (3.4 to 13.33) | 0.002 |
| Hawaii | 1999-2012 | -2.49 (-3.39 to -1.06) | 0.006 |
| Hawaii | 2012-2015 | -17.88 (-21.86 to -9.03) | <0.001 |
| Hawaii | 2015-2023 | 2.21 (-0.31 to 8.43) | 0.076 |
| Idaho | 1999-2018 | -2.44 (-9.17 to -1.24) | 0.027 |
| Idaho | 2018-2023 | 5.46 (-1.05 to 24.76) | 0.141 |
| Illinois | 1999-2012 | -2.57 (-3.44 to -0.39) | 0.043 |
| Illinois | 2012-2015 | -13.29 (-17.44 to -4.99) | 0.006 |
| Illinois | 2015-2023 | 2.65 (0.19 to 11.5) | 0.038 |
| Indiana | 1999-2023 | -0.92 (-1.75 to -0.09) | 0.033 |
| Iowa | 1999-2011 | 0.29 (-0.97 to 3.29) | 0.509 |
| Iowa | 2011-2014 | -13.01 (-17.08 to -3.75) | 0.002 |
| Iowa | 2014-2023 | 5.04 (3.04 to 8.58) | 0.002 |
| Kansas | 1999-2001 | 11.57 (-2.84 to 22.31) | 0.196 |
| Kansas | 2001-2016 | -3.8 (-8.99 to -2.03) | 0.043 |
| Kansas | 2016-2023 | 4.35 (0.68 to 10.13) | 0.032 |
| Kentucky | 1999-2012 | 0.2 (-1.14 to 5.97) | 0.569 |
| Kentucky | 2012-2015 | -13.52 (-19 to -2.46) | 0.016 |
| Kentucky | 2015-2023 | 7.91 (4.79 to 18.21) | 0.009 |
| Louisiana | 1999-2012 | -2.71 (-3.89 to 1.63) | 0.075 |
| Louisiana | 2012-2015 | -12.85 (-16.87 to -4.06) | 0.027 |
| Louisiana | 2015-2023 | 4.44 (1.74 to 9.63) | 0.022 |
| Maine | 1999-2003 | 1.6 (-3.23 to 12.18) | 0.565 |
| Maine | 2003-2016 | -5.12 (-11.39 to -4.17) | 0.014 |
| Maine | 2016-2023 | 0.72 (-2.29 to 7.02) | 0.600 |
| Maryland | 1999-2011 | -2.97 (-4.07 to -1.23) | 0.013 |
| Maryland | 2011-2014 | -19.09 (-23.17 to -9.66) | 0.001 |
| Maryland | 2014-2023 | 6.57 (4.43 to 10.17) | <0.001 |
| Massachusetts | 1999-2011 | -1.33 (-2.14 to 0.22) | 0.076 |
| Massachusetts | 2011-2014 | -10.86 (-13.77 to -4.42) | 0.003 |
| Massachusetts | 2014-2023 | 3.18 (1.78 to 5.58) | 0.002 |
| Michigan | 1999-2012 | -2.29 (-3.39 to 0.86) | 0.075 |
| Michigan | 2012-2015 | -13.83 (-18.22 to -4.69) | 0.010 |
| Michigan | 2015-2023 | 4.5 (1.81 to 10.83) | 0.010 |
| Minnesota | 1999-2012 | 0.47 (-1.02 to 5.38) | 0.394 |
| Minnesota | 2012-2015 | -14.01 (-19.62 to -2.94) | 0.009 |
| Minnesota | 2015-2023 | 5.53 (2.28 to 15.78) | 0.010 |
| Mississippi | 1999-2012 | -1.24 (-2.46 to 0.89) | 0.203 |
| Mississippi | 2012-2015 | -18.79 (-23.95 to -7.62) | <0.001 |
| Mississippi | 2015-2023 | 7.78 (4.53 to 14.36) | <0.001 |
| Missouri | 1999-2012 | -2.05 (-2.91 to -0.25) | 0.040 |
| Missouri | 2012-2015 | -13.4 (-17.22 to -5.1) | 0.005 |
| Missouri | 2015-2023 | 4.59 (2.31 to 9.35) | 0.004 |
| Montana | 1999-2018 | -2.51 (-3.75 to -1.1) | 0.034 |
| Montana | 2018-2021 | 19.82 (-4.38 to 26.94) | 0.111 |
| Montana | 2021-2023 | -10.63 (-20.52 to 7.96) | 0.160 |
| Nebraska | 1999-2011 | 2.03 (0.78 to 4.04) | 0.002 |
| Nebraska | 2011-2014 | -15.28 (-19.19 to -6.42) | <0.001 |
| Nebraska | 2014-2023 | 5.29 (3.39 to 8.55) | <0.001 |
| Nevada | 1999-2015 | -5.6 (-8.1 to -3.92) | <0.001 |
| Nevada | 2015-2023 | 8.5 (4.34 to 17.94) | <0.001 |
| New Hampshire | 1999-2012 | -0.76 (-2.37 to 4.3) | 0.566 |
| New Hampshire | 2012-2015 | -16.54 (-22.78 to -4.12) | 0.022 |
| New Hampshire | 2015-2023 | 4.36 (0.07 to 20.97) | 0.047 |
| New Jersey | 1999-2012 | -1.51 (-2.43 to -0.05) | 0.046 |
| New Jersey | 2012-2015 | -14.65 (-18.43 to -6.6) | <0.001 |
| New Jersey | 2015-2023 | 3.44 (1.24 to 7.72) | 0.003 |
| New Mexico | 1999-2011 | 0.96 (-0.29 to 3.02) | 0.124 |
| New Mexico | 2011-2014 | -16.75 (-20.76 to -7.61) | <0.001 |
| New Mexico | 2014-2023 | 2.28 (0.28 to 6.19) | 0.029 |
| New York | 1999-2012 | -2 (-2.97 to -0.44) | 0.028 |
| New York | 2012-2015 | -15.38 (-19.46 to -6.79) | 0.002 |
| New York | 2015-2023 | 1.71 (-0.77 to 8.8) | 0.146 |
| North Carolina | 1999-2012 | -1.66 (-2.69 to 0.3) | 0.076 |
| North Carolina | 2012-2015 | -15.37 (-19.55 to -6.29) | 0.002 |
| North Carolina | 2015-2023 | 5.02 (2.52 to 9.8) | 0.001 |
| North Dakota | 1999-2012 | 1.13 (-0.07 to 3.1) | 0.065 |
| North Dakota | 2012-2015 | -18.28 (-23.27 to -7.36) | <0.001 |
| North Dakota | 2015-2023 | 4.65 (1.55 to 12.87) | 0.003 |
| Ohio | 1999-2012 | -1.98 (-3.14 to -0.02) | 0.048 |
| Ohio | 2012-2015 | -19.51 (-24.63 to -8.48) | <0.001 |
| Ohio | 2015-2023 | 3.91 (0.57 to 12.97) | 0.024 |
| Oklahoma | 1999-2012 | -0.28 (-1.54 to 3.3) | 0.961 |
| Oklahoma | 2012-2015 | -13.64 (-18.5 to -3.42) | 0.008 |
| Oklahoma | 2015-2023 | 5.57 (2.71 to 12.2) | 0.006 |
| Oregon | 1999-2012 | 1.77 (0.54 to 4.69) | 0.008 |
| Oregon | 2012-2015 | -12.36 (-17.18 to -2.47) | 0.004 |
| Oregon | 2015-2023 | 6.12 (3.52 to 12.7) | 0.002 |
| Pennsylvania | 1999-2012 | -2.64 (-3.71 to 1.12) | 0.068 |
| Pennsylvania | 2012-2015 | -12.75 (-17.09 to -4.07) | 0.022 |
| Pennsylvania | 2015-2023 | 5.3 (2.62 to 11.23) | 0.015 |
| Rhode Island | 1999-2023 | -2.89 (-3.92 to -1.99) | <0.001 |
| South Carolina | 1999-2012 | -1.63 (-2.73 to 4.31) | 0.144 |
| South Carolina | 2012-2015 | -9.91 (-13.81 to -2.56) | 0.030 |
| South Carolina | 2015-2023 | 3.27 (0.88 to 9.81) | 0.029 |
| South Dakota | 1999-2011 | 0.78 (-0.55 to 3.66) | 0.193 |
| South Dakota | 2011-2014 | -13.1 (-17.64 to -3.07) | 0.009 |
| South Dakota | 2014-2023 | 6.63 (4.33 to 11.18) | 0.005 |
| Tennessee | 1999-2012 | -1.09 (-2.38 to 2.21) | 0.323 |
| Tennessee | 2012-2015 | -16.09 (-21.58 to -4.58) | 0.009 |
| Tennessee | 2015-2023 | 5.24 (1.85 to 18.17) | 0.013 |
| Texas | 1999-2023 | -1.18 (-2.02 to -0.28) | 0.009 |
| Utah | 1999-2015 | -1.03 (-3.77 to 0.03) | 0.056 |
| Utah | 2015-2023 | 4.62 (2.11 to 12.74) | <0.001 |
| Vermont | 1999-2012 | -0.16 (-1.6 to 2.11) | 0.999 |
| Vermont | 2012-2016 | -16.5 (-25.31 to -8.16) | <0.001 |
| Vermont | 2016-2023 | 5.28 (0.96 to 16.29) | 0.020 |
| Virginia | 1999-2012 | -2.15 (-4.25 to 11.12) | 0.178 |
| Virginia | 2012-2015 | -13.38 (-19.64 to 7.27) | 0.070 |
| Virginia | 2015-2023 | 6.94 (1.06 to 21) | 0.048 |
| Washington | 1999-2012 | 0.98 (-0.42 to 4.35) | 0.140 |
| Washington | 2012-2016 | -12.35 (-21.16 to -3.99) | 0.008 |
| Washington | 2016-2023 | 6.4 (2.26 to 21.84) | 0.011 |
| West Virginia | 1999-2012 | -1.32 (-3.05 to 4.97) | 0.340 |
| West Virginia | 2012-2015 | -17.17 (-24.11 to -4.36) | 0.027 |
| West Virginia | 2015-2023 | 4.5 (-0.52 to 23.61) | 0.062 |
| Wisconsin | 1999-2017 | -1.58 (-3.82 to -0.69) | 0.002 |
| Wisconsin | 2017-2023 | 6.15 (1.51 to 18.98) | 0.003 |
| Wyoming | 1999-2018 | -1.61 (-2.81 to -0.88) | 0.001 |
| Wyoming | 2018-2023 | 6.59 (1.84 to 18.54) | 0.005 |

| **Supplementary Table 9. Sensitivity analyses for county-level suppressed death counts.** | | | |
| --- | --- | --- | --- |
| **Metric** | **Comparison** | **Spearman correlation with primary analysis** | **LISA same-cluster percentage** |
| Stage 4 AAMR | deaths = 1 vs deaths = 5 | 0.794 | 91.80% |
| Stage 4 AAMR | deaths = 9 vs deaths = 5 | 0.943 | 94.00% |
| Stage 4b AAMR | deaths = 1 vs deaths = 5 | 0.251 | 68.30% |
| Stage 4b AAMR | deaths = 9 vs deaths = 5 | 0.947 | 89.00% |
| Stage 4b share | deaths = 1 vs deaths = 5 | 0.271 | 73.90% |
| Stage 4b share | deaths = 9 vs deaths = 5 | 0.906 | 92.90% |
| The primary analysis assigned suppressed death counts a value of 5. Sensitivity analyses assigned suppressed cells values of 1 and 9. Spearman correlations compare county-level metric rankings with the primary analysis. LISA same-cluster percentage represents the proportion of counties assigned to the same LISA category as in the primary analysis among counties included in LISA analyses. AAMR, age-adjusted mortality rate; LISA, Local Indicators of Spatial Association. | | | |

# **Supplementary Figures**


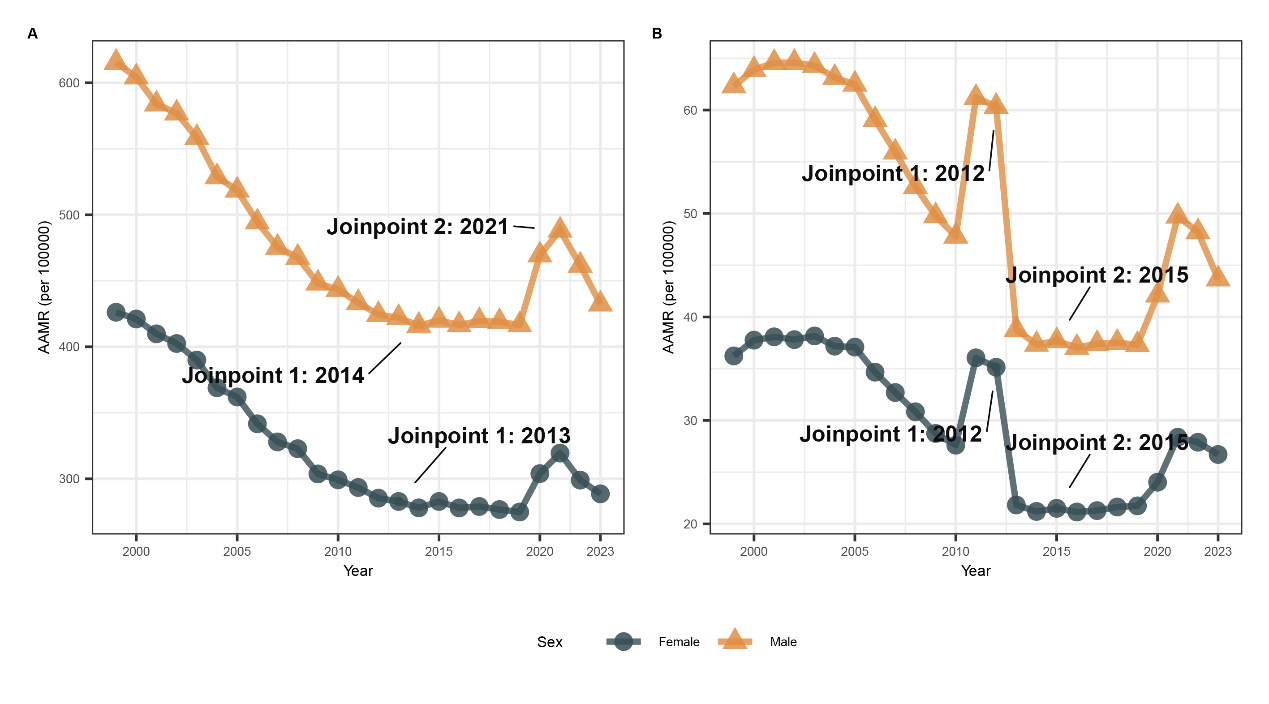


## **Supplementary Figure 1. Temporal trends in AAMR for Stage 4 (A) and Stage 4b (B) CKM syndrome by sex, 1999–2023.**

AAPC: average annual percent change, AAMR: age-adjusted mortality rates, CKM: cardiovascular–kidney–metabolic.


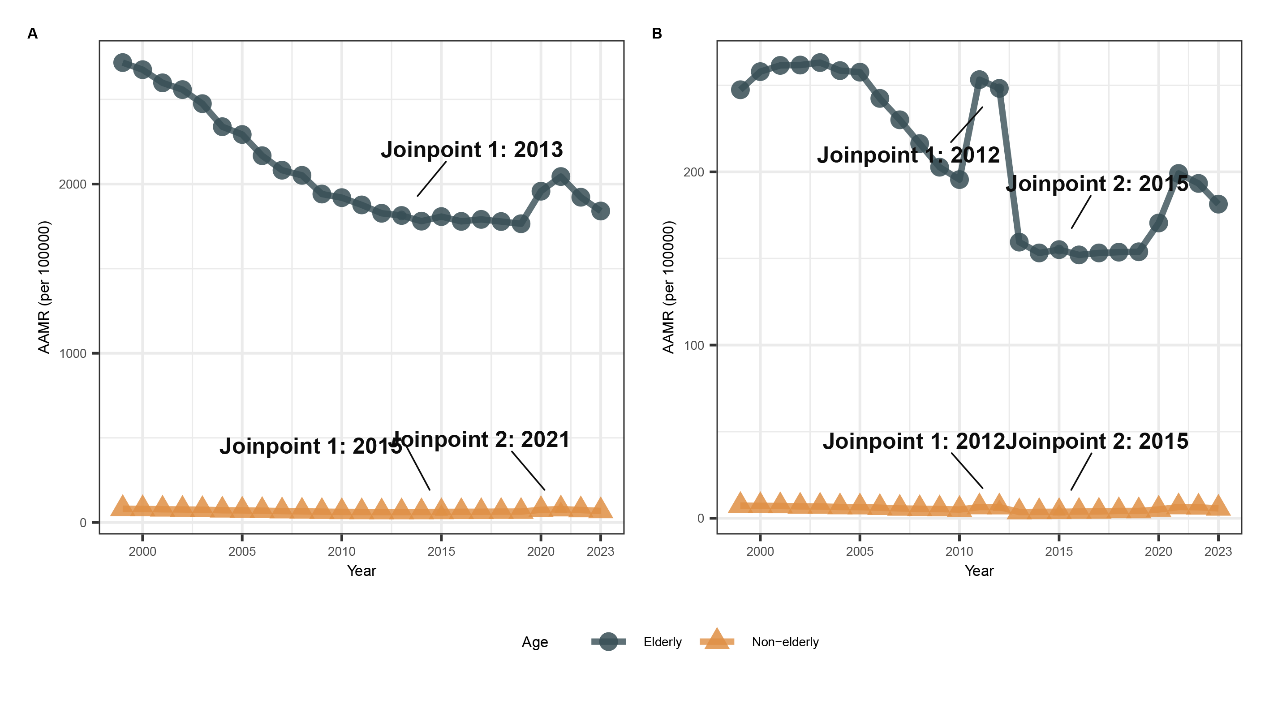


## **Supplementary Figure 2. Temporal trends in AAMR for Stage 4 (A) and Stage 4b (B) CKM syndrome by age, 1999–2023.**

AAPC: average annual percent change, AAMR: age-adjusted mortality rates, CKM: cardiovascular–kidney–metabolic.


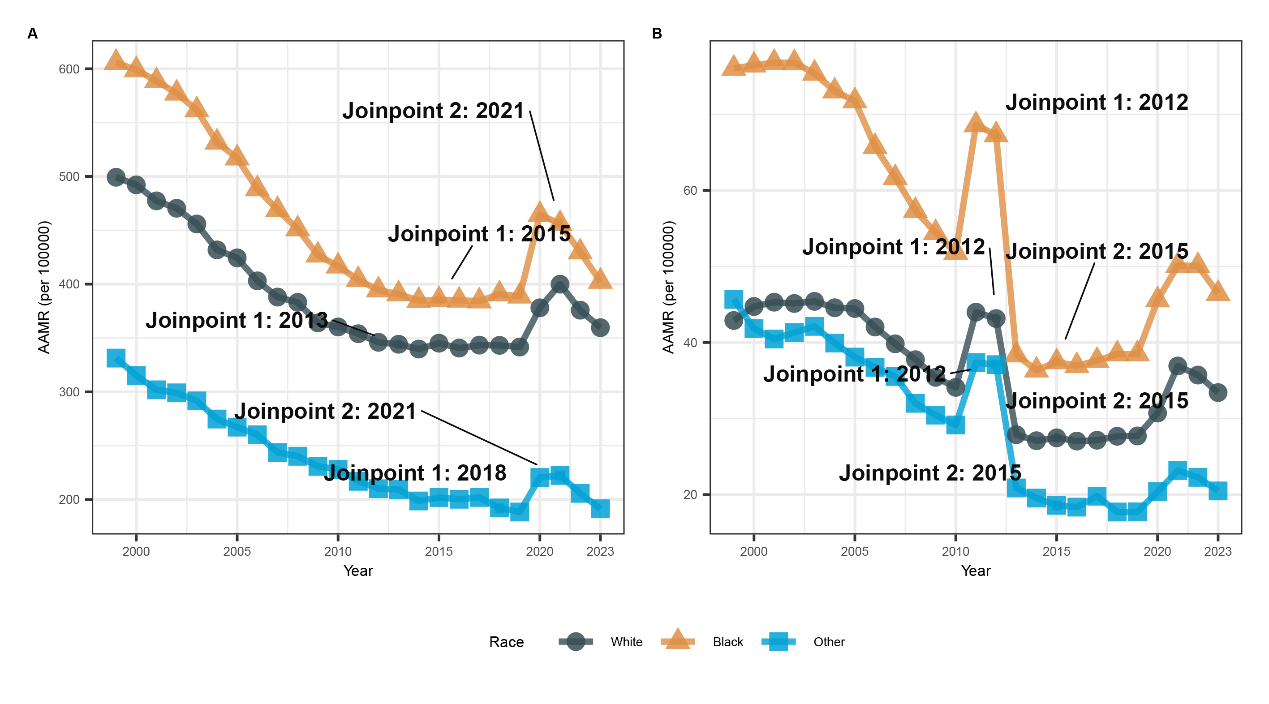


## **Supplementary Figure 3. Temporal trends in AAMR for Stage 4 (A) and Stage 4b (B) CKM syndrome by race, 1999–2023.**

AAPC: average annual percent change, AAMR: age-adjusted mortality rates, CKM: cardiovascular–kidney–metabolic.


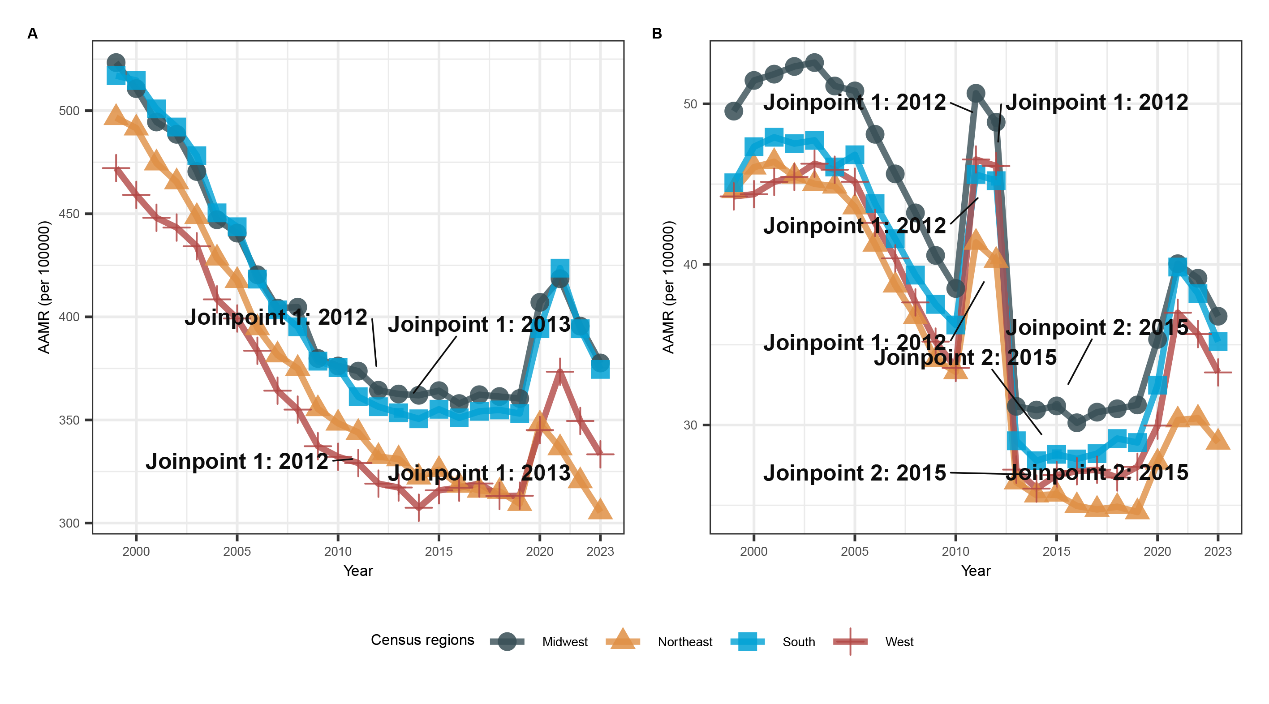


## **Supplementary Figure 4. Temporal trends in AAMR for Stage 4 (A) and Stage 4b (B) CKM syndrome by census regions, 1999–2023.**

AAPC: average annual percent change, AAMR: age-adjusted mortality rates, CKM: cardiovascular–kidney–metabolic.


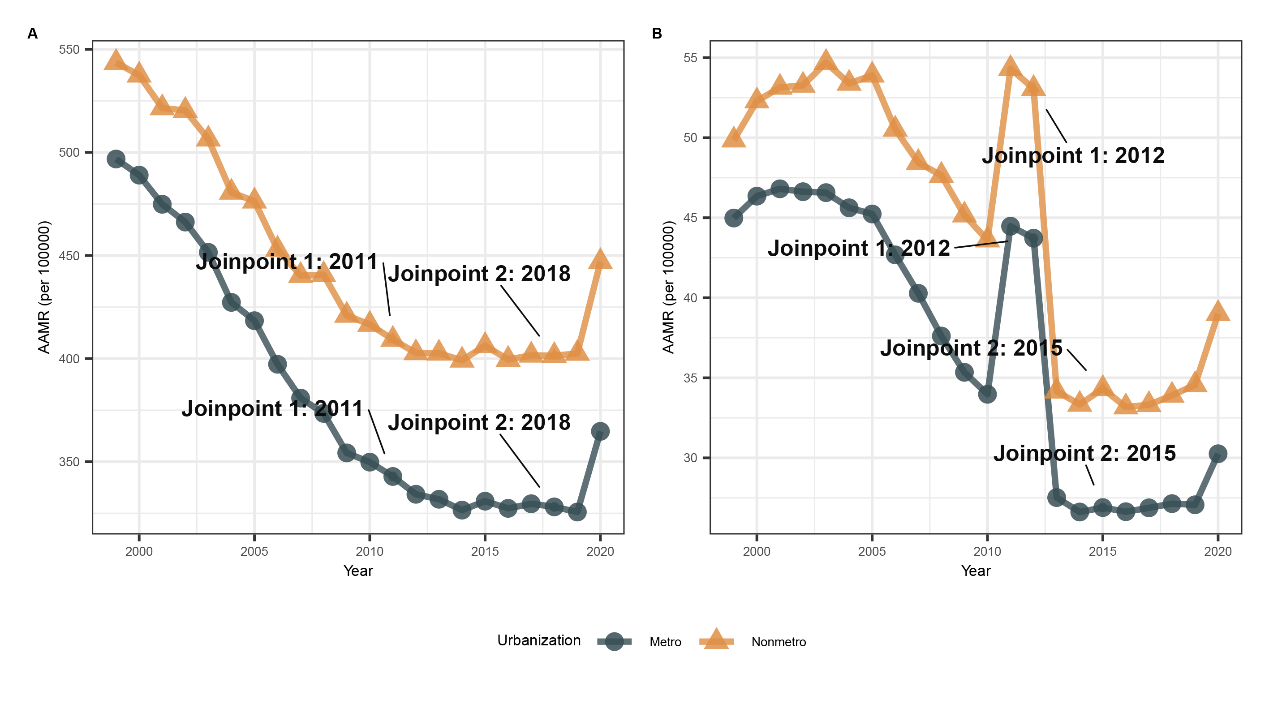


## **Supplementary Figure 5. Temporal trends in AAMR for Stage 4 (A) and Stage 4b (B) CKM syndrome by urbanization, 1999–2020.**

AAPC: average annual percent change, AAMR: age-adjusted mortality rates, CKM: cardiovascular–kidney–metabolic.


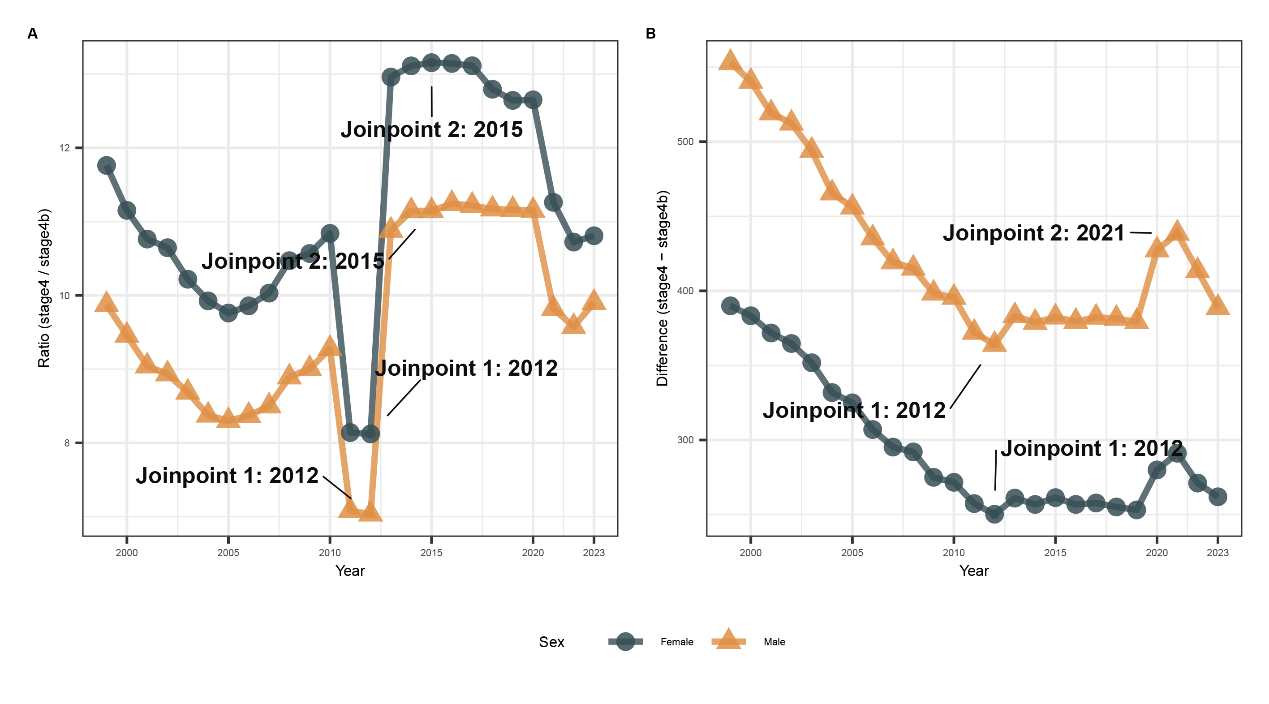


## **Supplementary Figure 6. Temporal trends in the AAMR ratio and absolute rate difference for CKM syndrome by sex, 1999–2023.**

AAMR: age-adjusted mortality rates, CKM: cardiovascular–kidney–metabolic.


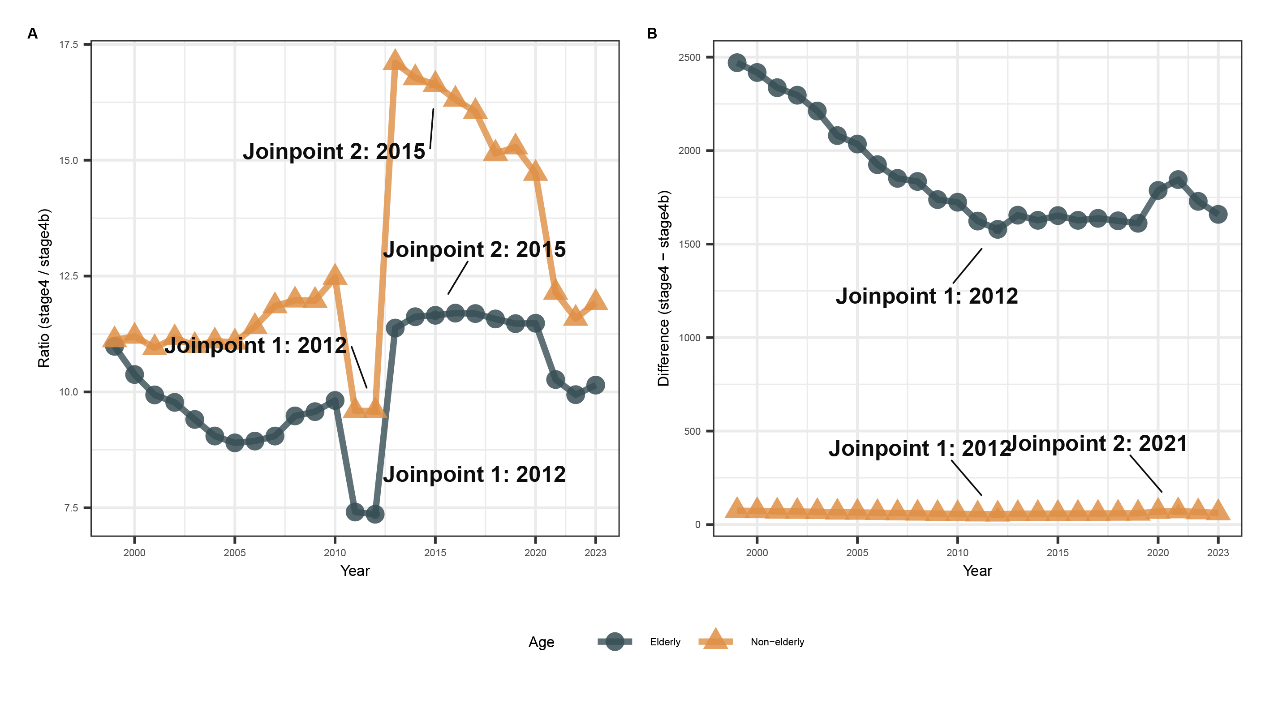


## **Supplementary Figure 7. Temporal trends in the AAMR ratio and absolute rate difference for CKM syndrome by age, 1999–2023.**

AAMR: age-adjusted mortality rates, CKM: cardiovascular–kidney–metabolic.


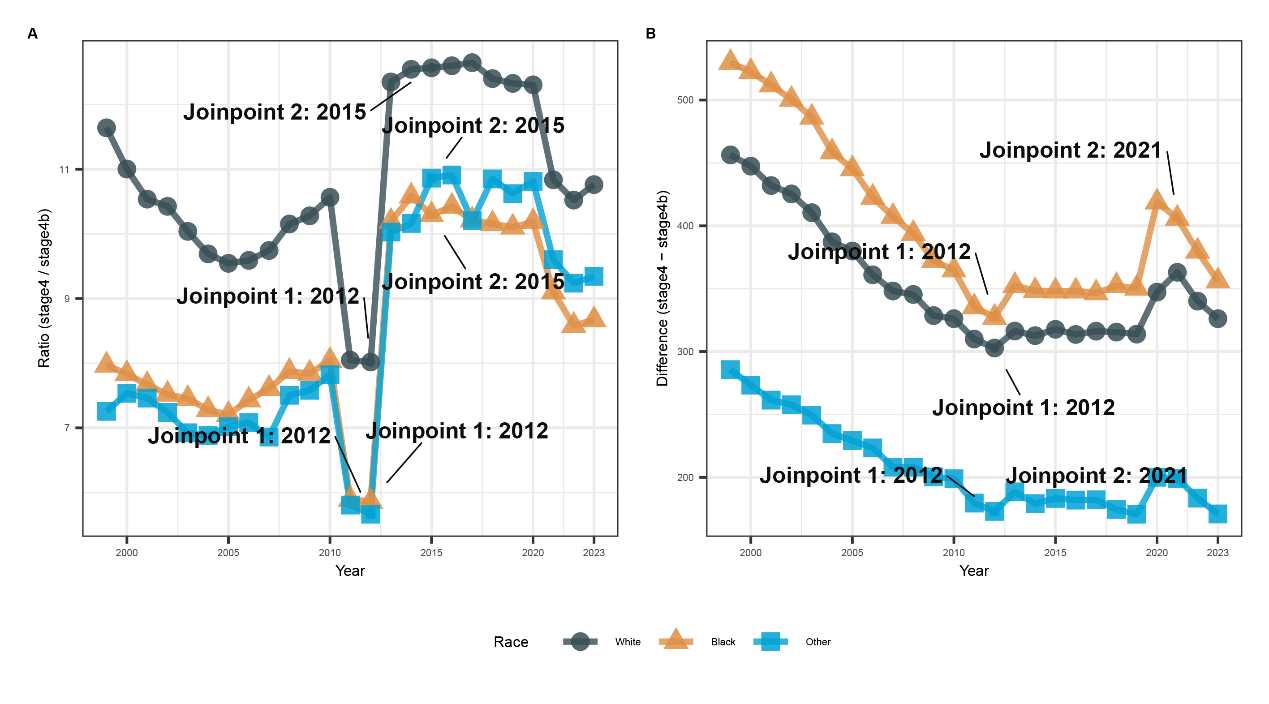


## **Supplementary Figure 8. Temporal trends in the AAMR ratio and absolute rate difference for CKM syndrome by race, 1999–2023.**

AAMR: age-adjusted mortality rates, CKM: cardiovascular–kidney–metabolic.


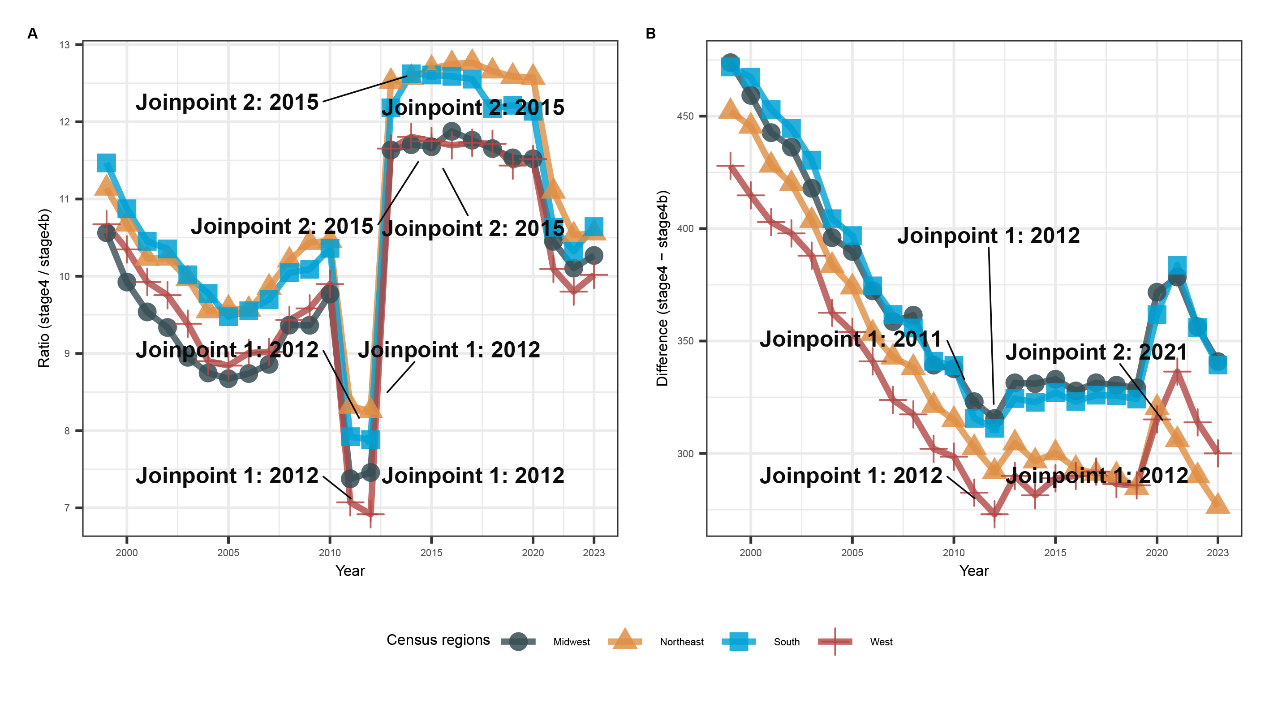


## **Supplementary Figure 9. Temporal trends in the AAMR ratio and absolute rate difference for CKM syndrome by census regions, 1999–2023.**

AAMR: age-adjusted mortality rates, CKM: cardiovascular–kidney–metabolic.


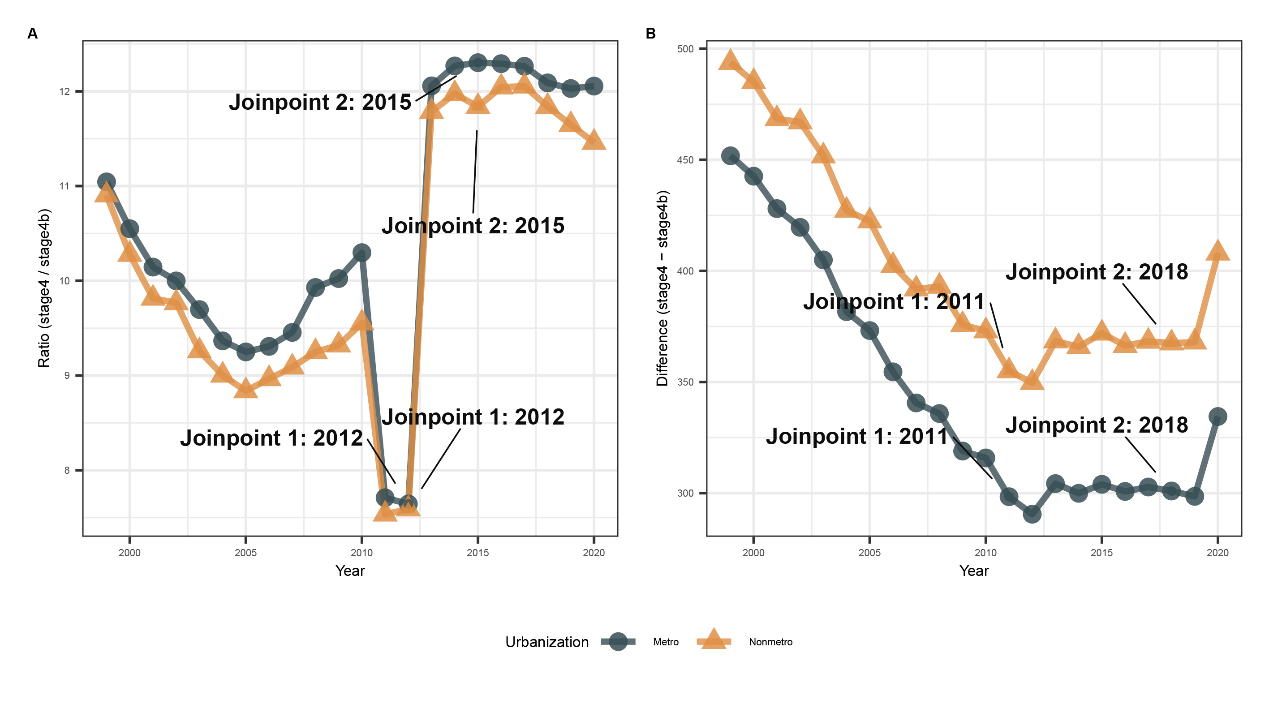


## **Supplementary Figure 10. Temporal trends in the AAMR ratio and absolute rate difference for CKM syndrome by urbanization, 1999–2020.**

AAMR: age-adjusted mortality rates, CKM: cardiovascular–kidney–metabolic.


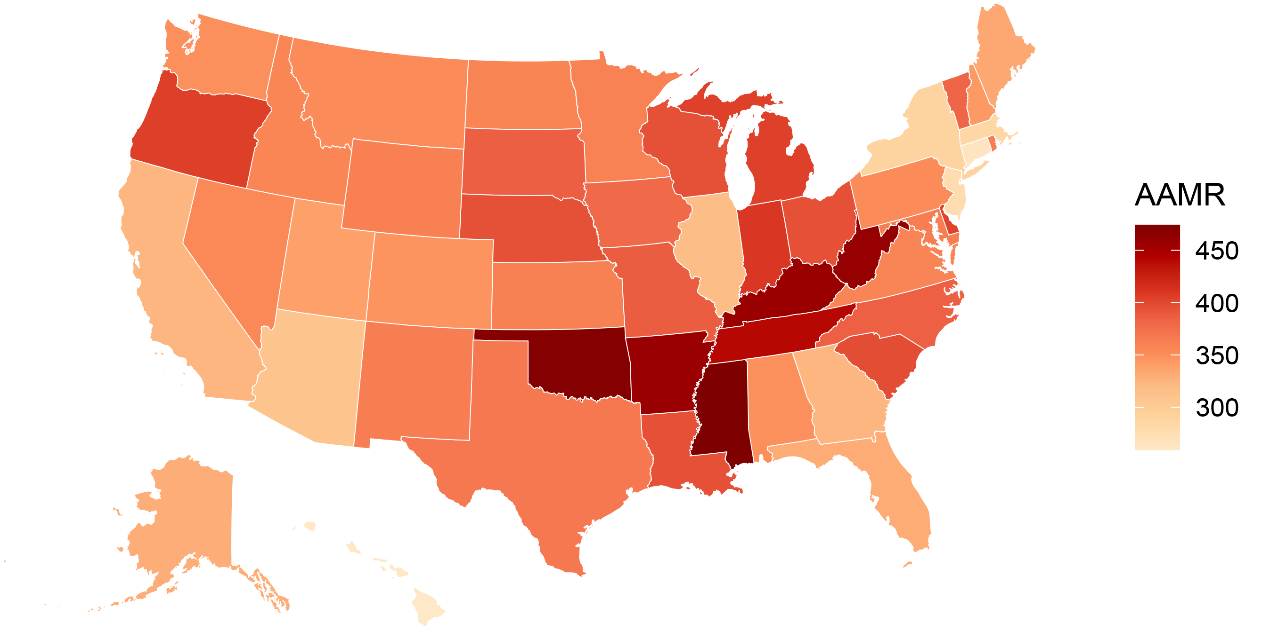


## **Supplementary Figure 11. State-level distribution of AAMR for CKM Stage 4, 2023.**

AAMR: age-adjusted mortality rates, CKM: cardiovascular–kidney–metabolic.


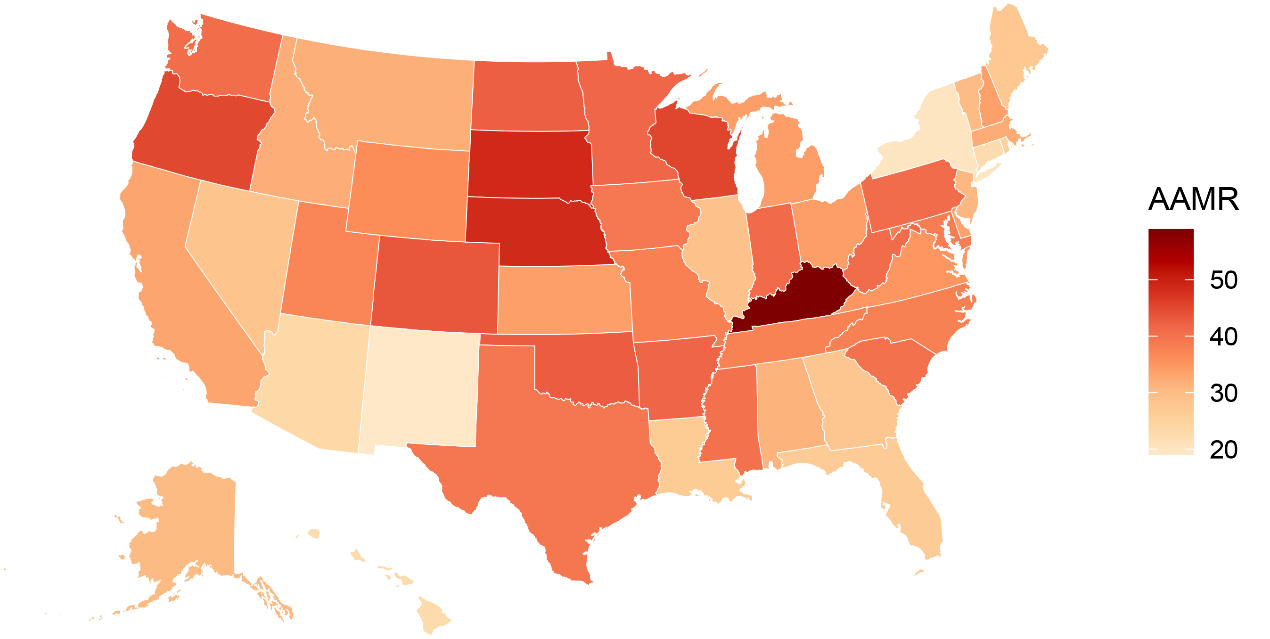


## **Supplementary Figure 12. State-level distribution of AAMR for CKM Stage 4b, 2023.**

AAMR: age-adjusted mortality rates, CKM: cardiovascular–kidney–metabolic.


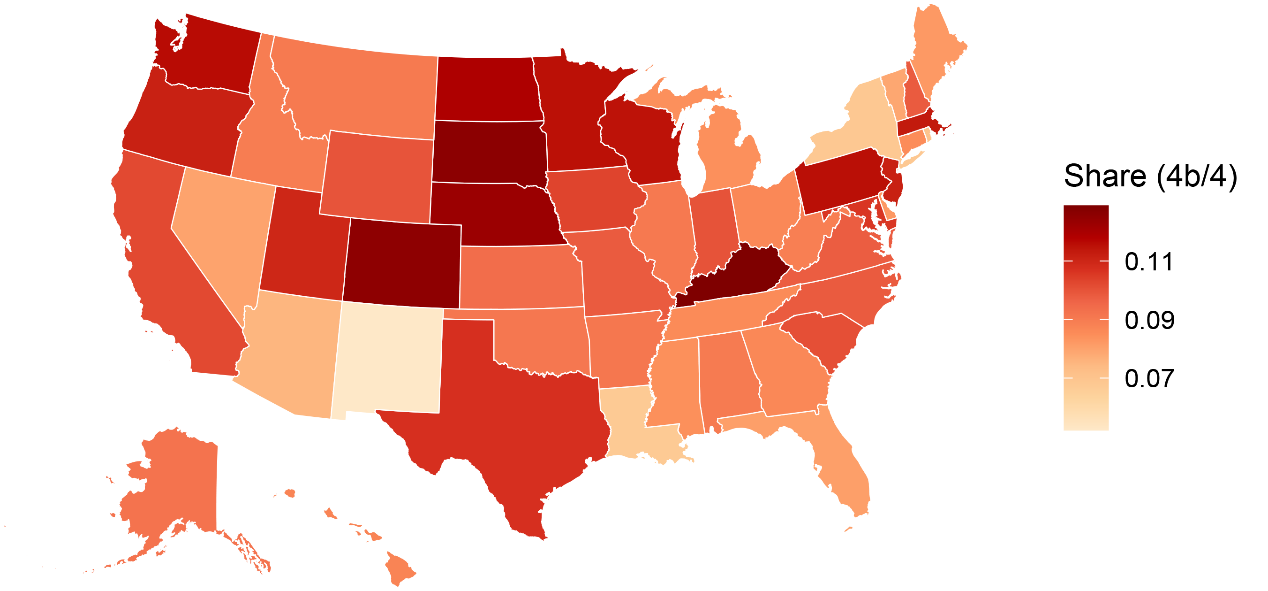


## **Supplementary Figure 13. State-level variation in the proportional contribution (share) of CKM Stage 4b within Stage 4 (4b/4), 2023.**

AAMR: age-adjusted mortality rates, CKM: cardiovascular–kidney–metabolic.


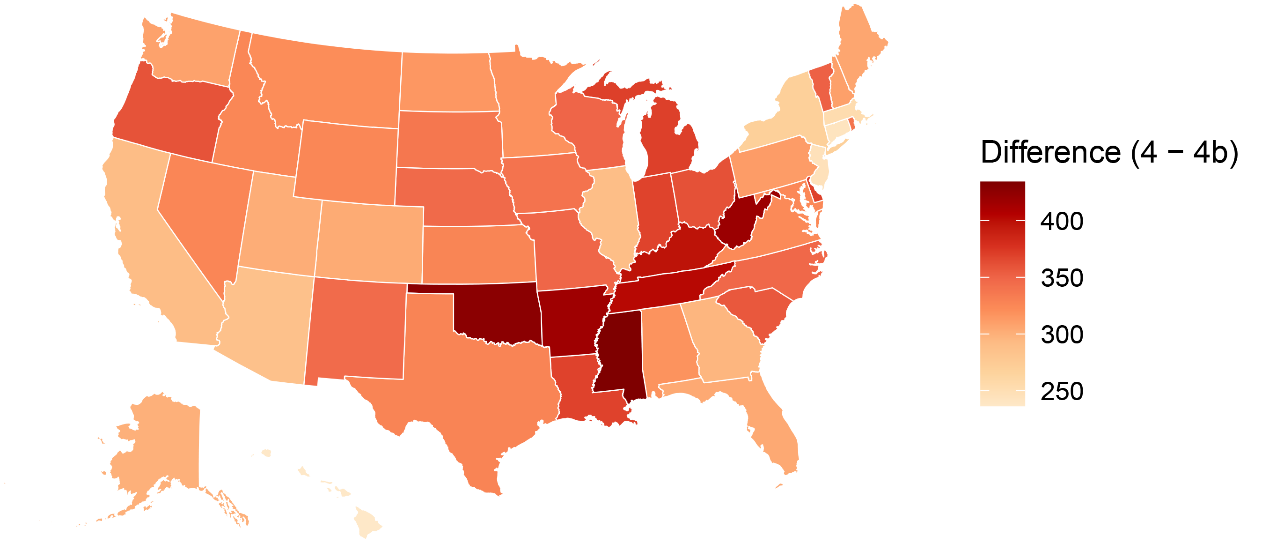


## **Supplementary Figure 14. State-level distribution of the absolute AAMR gap between CKM Stage 4 and Stage 4b (Stage 4 − Stage 4b), 2023.**

AAMR: age-adjusted mortality rates, CKM: cardiovascular–kidney–metabolic.


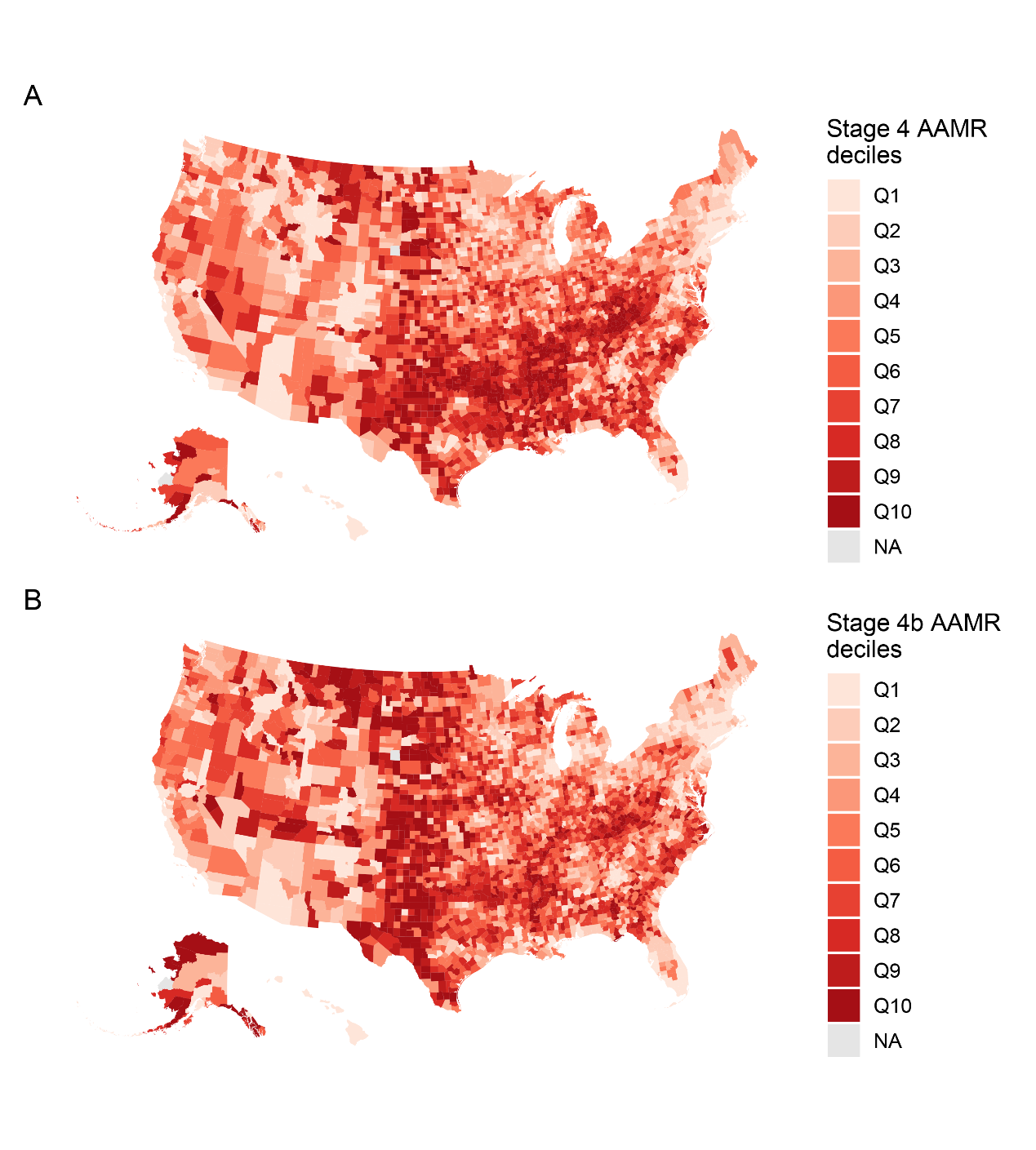


## **Supplementary Figure 15. County-level geographic distribution (deciles) of age-adjusted mortality rates for CKM Stage 4 (A) and Stage 4b (B), 2023.**

AAMR was directly standardized to the 2000 US standard population for ages 15–84 years.

AAMR: age-adjusted mortality rates, CKM: cardiovascular–kidney–metabolic.


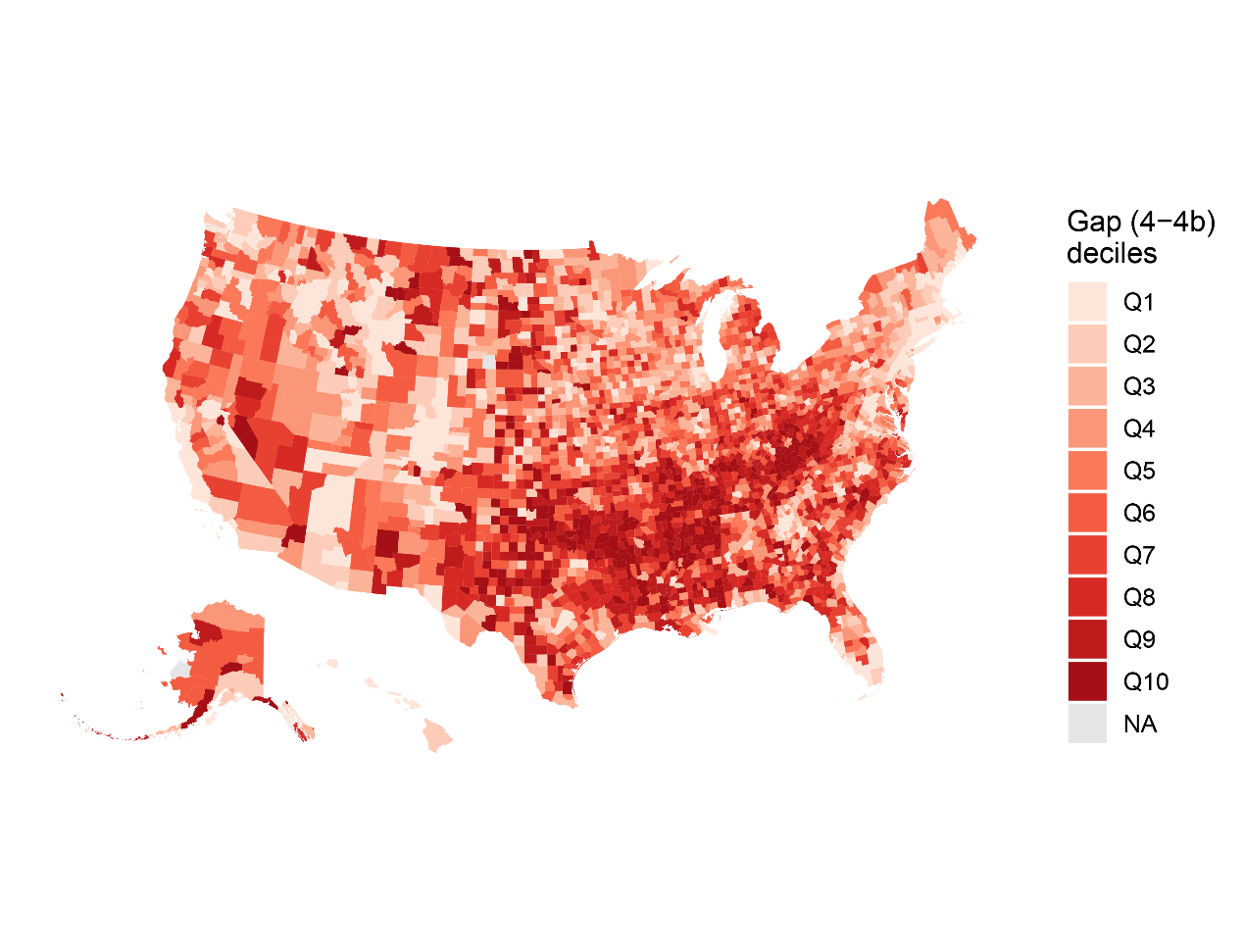


## **Supplementary Figure 16. County-level geographic distribution (deciles) of the absolute AAMR gap between CKM Stage 4 and Stage 4b (Stage 4 − Stage 4b), 2023.**

AAMR: age-adjusted mortality rates, CKM: cardiovascular–kidney–metabolic.


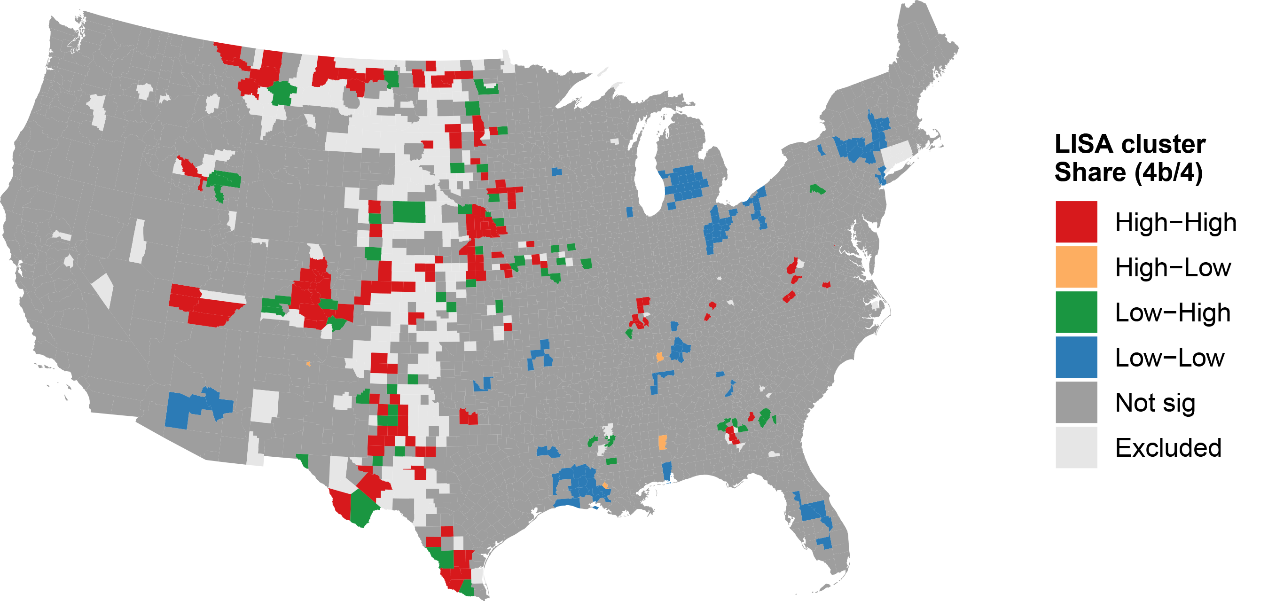


## **Supplementary Figure 17. LISA cluster map for the Stage 4b share within CKM Stage 4 (4b/4), 2023.**

LISA was estimated for contiguous US counties with population (ages 15–84) ≥20,000.

LISA: local indicators of spatial association, CKM, cardiovascular–kidney–metabolic.
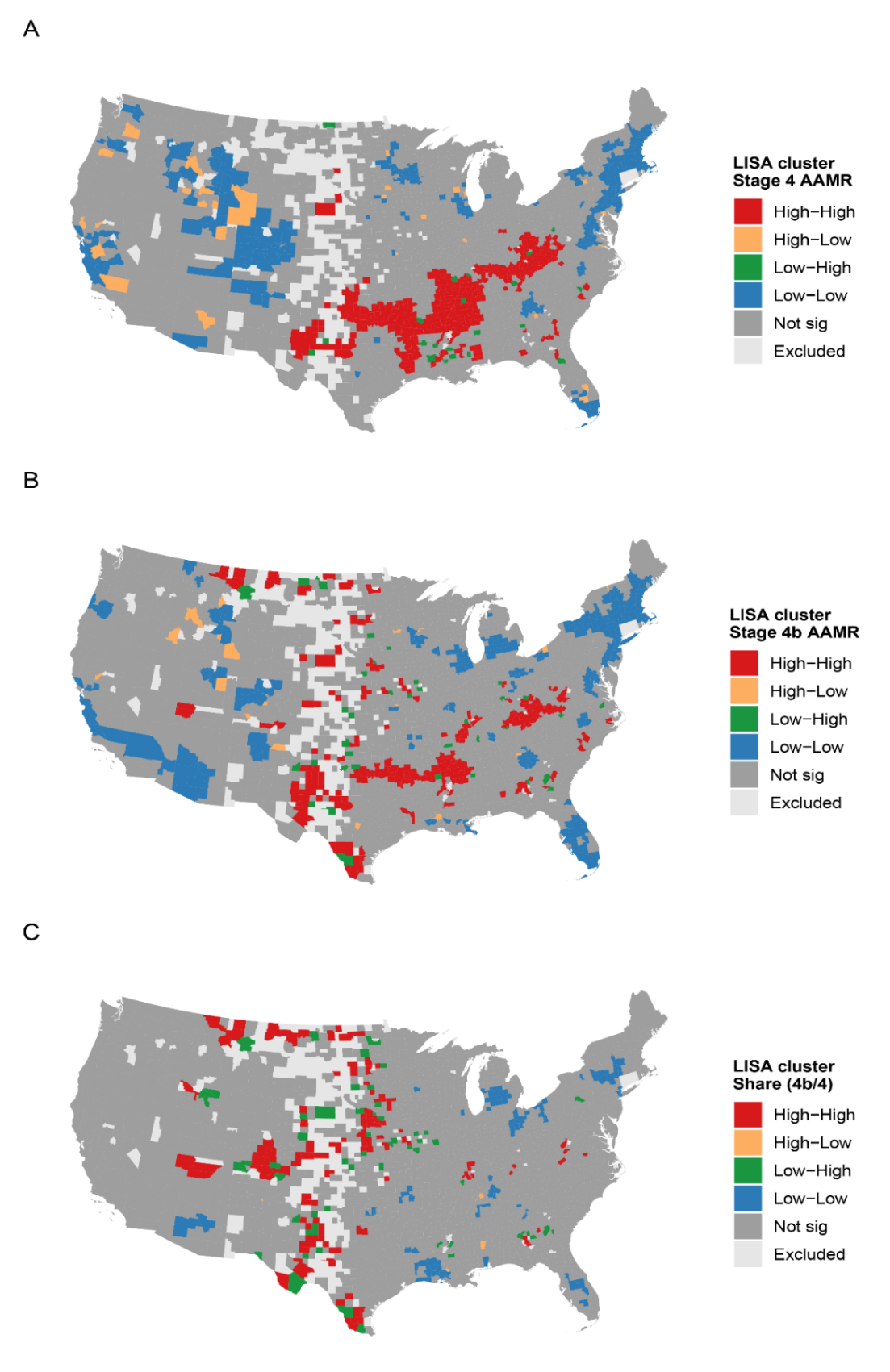


## **Supplementary Figure 18. County-level LISA cluster maps for CKM age-adjusted mortality rates for Stage 4 (A), Stage 4b (B), and the Stage 4b share within Stage 4 (4b/4) (C), 2023.**

LISA was estimated for contiguous US counties with population (ages 15–84) ≥20,000.

CKM, cardiovascular–kidney–metabolic; LISA, local indicators of spatial association; AAMR, age-adjusted mortality rate.
