# Supplementary material for: National trends and county-level geographic disparities in mortality from operationally defined cardiovascular–kidney–metabolic stages 4 and 4b in the United States
Source: Front Cardiovasc Med. 2026 May 29;13:1838375. doi: 10.3389/fcvm.2026.1838375 (PMC13259836; doi:10.3389/fcvm.2026.1838375)
Supplement: Supplementary file 2 [file Datasheet2.docx]

STROBE Statement—checklist of items that should be included in reports of observational studies

|  | Item No. | Recommendation | Location in manuscript | Relevant text from manuscript |
| --- | --- | --- | --- | --- |
| **Title and abstract** | 1 | (*a*) Indicate the study’s design with a commonly used term in the title or the abstract | \|  \| \| --- \|  \| Title; Abstract: Methods \| \| --- \| | Title: "National Trends and County-Level Geographic Disparities in Mortality From Operationally Defined…" ; Methods: "We conducted an ecological time-series and spatial analysis of US mortality data from 1999 to 2023…" |
|  |  | (*b*) Provide in the abstract an informative and balanced summary of what was done and what was found | Abstract | Abstract: "Using CDC WONDER multiple-cause-of-death data (1999–2023), we calculated age-adjusted mortality rates (AAMRs)… applied joinpoint regression… mapped state- and county-level AAMRs… and assessed local spatial clustering (LISA)…" |
| Introduction | | | |  |
| Background/rationale | 2 | Explain the scientific background and rationale for the investigation being reported | Introduction | Introduction: "CKM syndrome has emerged as a unifying framework… Stage 4 represents… Stage 4b… population-level evidence on long-term mortality trends and geographic inequities… remains limited." |
| Objectives | 3 | State specific objectives, including any prespecified hypotheses | Introduction | Introduction: "To address these gaps, we examined US mortality rates for CKM Stage 4 and Stage 4b from 1999 to 2023… quantified temporal patterns using joinpoint regression… characterized state- and county-level geographic disparities… and quantified county-level spatial clustering (LISA)." |
| Methods | | | |  |
| Study design | 4 | Present key elements of study design early in the paper | Methods: Data source | Methods: "We conducted an ecological time-series and spatial analysis of US mortality data from 1999 to 2023…" |
| Setting | 5 | Describe the setting, locations, and relevant dates, including periods of recruitment, exposure, follow-up, and data collection | Methods: Data source | Methods (Data source): "US mortality data from 1999 to 2023… CDC WONDER Multiple Cause of Death database… death certificates for United States residents…" |
| Participants | 6 | (*a*) *Cohort study*—Give the eligibility criteria, and the sources and methods of selection of participants. Describe methods of follow-up  *Case-control study*—Give the eligibility criteria, and the sources and methods of case ascertainment and control selection. Give the rationale for the choice of cases and controls  *Cross-sectional study*—Give the eligibility criteria, and the sources and methods of selection of participants | Methods: Data source; Methods: Spatial analysis | Methods: "The database compiles information from death certificates for United States residents…" ; County analyses: "Analyses were restricted to the contiguous United States and to counties with nonmissing values and total population (ages 15–84 years) ≥20,000." |
|  |  | (*b*) *Cohort study*—For matched studies, give matching criteria and number of exposed and unexposed  *Case-control study*—For matched studies, give matching criteria and the number of controls per case | N/A | Not applicable (ecological, aggregated mortality data; no matching). |
| Variables | 7 | Clearly define all outcomes, exposures, predictors, potential confounders, and effect modifiers. Give diagnostic criteria, if applicable | Methods: CKM Stage 4/4b ascertainment | Methods (Ascertainment): "Stage 4 was defined by… ICD-10 codes… I20–I25, I50, I60–I69, I48, or I70… Stage 4b was defined as Stage 4 with concurrent kidney failure… N17–N19." |
| Data sources/ measurement | 8* | For each variable of interest, give sources of data and details of methods of assessment (measurement). Describe comparability of assessment methods if there is more than one group | Methods: Data source; Methods: CKM Stage 4/4b ascertainment | Methods (Data source/measurement): "CDC WONDER… provides counts, age-adjusted rates, standard errors/95% CIs, and both underlying and contributing causes of death…"; plus ICD-10 code ascertainment described in Section 2.2. |
| Bias | 9 | Describe any efforts to address potential sources of bias | Methods: Spatial analysis; Discussion: Limitations | Limitations: "misclassification and temporal changes in certification or coding practices may affect trends…"; "Suppressed death counts…"; "LISA… many simultaneous local tests… sensitivity to spatial weights…" |
| Study size | 10 | Explain how the study size was arrived at | Methods: Data source | Methods: "…US mortality data from 1999 to 2023… CDC WONDER…" (population-level analysis of all eligible death records; no a priori sample size calculation). |

Continued on next page

| Quantitative variables | 11 | Explain how quantitative variables were handled in the analyses. If applicable, describe which groupings were chosen and why | Methods: CKM Stage 4/4b ascertainment; Methods: Spatial analysis | Methods: "AAMRs per 100,000… standardized to the 2000 US standard population… constructed ratio (Stage 4/Stage 4b), share (Stage 4b/Stage 4), and absolute difference (Stage 4−Stage 4b)…"; "log transformation… deciles (Q1–Q10)." |
| --- | --- | --- | --- | --- |
| Statistical methods | 12 | (*a*) Describe all statistical methods, including those used to control for confounding | Methods: Trend analysis and uncertainty estimation; Methods: Spatial analysis | Methods (Trend/uncertainty): "approximated SEs… delta-method… Joinpoint regression… log-linear form… maximum of two joinpoints…"; Spatial methods described in Section 2.4. |
|  |  | (*b*) Describe any methods used to examine subgroups and interactions | Methods: Stratified analyses; Results: National and stratified temporal trends | Methods: "stratified by sex… age group… race… urbanization… Census region…"; Results report subgroup trends and geographic analyses. |
|  |  | (*c*) Explain how missing data were addressed | Methods: Spatial analysis | County methods: "Suppressed death counts were handled using midpoint substitution (deaths = 5)"; LISA restricted to counties with nonmissing values; exclusions specified. |
|  |  | (*d*) *Cohort study*—If applicable, explain how loss to follow-up was addressed  *Case-control study*—If applicable, explain how matching of cases and controls was addressed  *Cross-sectional study*—If applicable, describe analytical methods taking account of sampling strategy | N/A | Not applicable (no follow-up/loss to follow-up; not a cohort/case-control study). |
|  |  | (*e*) Describe any sensitivity analyses | Methods: Spatial analysis; Supplementary Table 9 | Sensitivity analyses were conducted by assigning suppressed death counts values of 1 and 9, compared with the primary assignment of 5; county-level rate estimation, decile mapping, and LISA analyses were repeated. |
| Results | | | | |
| Participants | 13* | (a) Report numbers of individuals at each stage of study—eg numbers potentially eligible, examined for eligibility, confirmed eligible, included in the study, completing follow-up, and analysed | N/A | Not applicable (no individual recruitment/flow; analysis used aggregated death certificate data). County analytic restrictions and exclusions are described in Section 2.4. |
|  |  | (b) Give reasons for non-participation at each stage | N/A | Not applicable (no participant enrollment). |
|  |  | (c) Consider use of a flow diagram | N/A | Not applicable (no flow diagram for aggregated mortality data). |
| Descriptive data | 14* | (a) Give characteristics of study participants (eg demographic, clinical, social) and information on exposures and potential confounders | Methods: Subgroup definitions; Results: National and stratified temporal trends; Table 1 | Methods: subgroup strata defined (sex, age group, race, urbanization, Census region); Results/Table 1 report stratified AAPCs and CIs ("Table 1. AAPC in national and subgroup AAMR…"). |
|  |  | (b) Indicate number of participants with missing data for each variable of interest | Methods: Spatial analysis | Methods: "Suppressed death counts were handled using midpoint substitution (deaths = 5)" and counties with nonmissing values were required for LISA. |
|  |  | (c) *Cohort study*—Summarise follow-up time (eg, average and total amount) | N/A | Not applicable (no cohort follow-up time). |
| Outcome data | 15* | *Cohort study*—Report numbers of outcome events or summary measures over time | Results: National and stratified temporal trends; Table 1 | Results/Table 1: "Stage 4 AAMR decreased… Stage 4b AAMR decreased…" and joinpoint APC/AAPC estimates with CIs are reported. |
|  |  | *Case-control study—*Report numbers in each exposure category, or summary measures of exposure | Results: National and stratified temporal trends; Table 1; Figures 1–2 | Results/Table 1: outcome measures (AAMR, APC/AAPC with 95% CIs) are reported for the study period. |
|  |  | *Cross-sectional study—*Report numbers of outcome events or summary measures | Results: National and stratified temporal trends; Table 1; Figures 1–4 | Results/Table 1 and Figures: outcome summary measures (AAMR, APC/AAPC with 95% CIs; 2023 state/county maps; LISA clusters). |
| Main results | 16 | (*a*) Give unadjusted estimates and, if applicable, confounder-adjusted estimates and their precision (eg, 95% confidence interval). Make clear which confounders were adjusted for and why they were included | \|  \| \| --- \|  \| Results: National and stratified temporal trends; Table 1 \| \| --- \| | Results: "AAMR decreased from… to…" ; "AAPC of −1.31… and −1.18…" ; Joinpoint segment APCs with P values are reported (no multivariable confounder adjustment beyond age-standardization). |
|  |  | (*b*) Report category boundaries when continuous variables were categorized | Methods: CKM Stage 4/4b ascertainment; Methods: Subgroup definitions | Methods: age group (15–64 vs ≥65), race categories (White, Black, Other), Metro vs Nonmetro, Census regions (NE, MW, South, West) defined. |
|  |  | (*c*) If relevant, consider translating estimates of relative risk into absolute risk for a meaningful time period | Results: National and stratified temporal trends; Results: Trends in Stage 4–Stage 4b relationship | Absolute measures are reported (AAMR per 100,000 and absolute rate difference Stage 4−Stage 4b). |

Continued on next page

| Other analyses | 17 | Report other analyses done—eg analyses of subgroups and interactions, and sensitivity analyses | Results: State-level geographic patterns; Results: County-level patterns and spatial clustering; Supplementary Tables/Figures | Other analyses: state- and county-level mapping; derived metrics (ratio, gap, share); LISA clustering; stratified analyses and state-level joinpoint models. |
| --- | --- | --- | --- | --- |
| Discussion | | | | |
| Key results | 18 | Summarise key results with reference to study objectives | \|  \| \| --- \|  \| Discussion \| \| --- \| | Discussion: "we identified three key findings…" summarizing national trends, inflection points, and geographic inequities. |
| Limitations | 19 | Discuss limitations of the study, taking into account sources of potential bias or imprecision. Discuss both direction and magnitude of any potential bias | Discussion: Limitations | Discussion (Limitations): "several limitations warrant consideration…" including misclassification, staging limitations, county AAMR construction/suppression handling, and LISA/multiple testing considerations. |
| Interpretation | 20 | Give a cautious overall interpretation of results considering objectives, limitations, multiplicity of analyses, results from similar studies, and other relevant evidence | Discussion | Discussion: interpretation of early-2010s transition and geographic patterns, with hypothesis-generating explanations and caution due to ecological design. |
| Generalisability | 21 | Discuss the generalisability (external validity) of the study results | \|  \| \| --- \|  \| Discussion: Limitations \| \| --- \| | Generalisability: findings pertain to US residents/death certificate data; authors note ecological design and staging limitations that may affect interpretation and applicability. |
| Other information | |  | | |
| Funding | 22 | Give the source of funding and the role of the funders for the present study and, if applicable, for the original study on which the present article is based | Acknowledgements | Acknowledgements: “This study received no external funding.” |

*Give information separately for cases and controls in case-control studies and, if applicable, for exposed and unexposed groups in cohort and cross-sectional studies.

**Note:** An Explanation and Elaboration article discusses each checklist item and gives methodological background and published examples of transparent reporting. The STROBE checklist is best used in conjunction with this article (freely available on the Web sites of PLoS Medicine at http://www.plosmedicine.org/, Annals of Internal Medicine at http://www.annals.org/, and Epidemiology at http://www.epidem.com/). Information on the STROBE Initiative is available at www.strobe-statement.org.
